# Identification of a membrane-less compartment regulating invadosome function and motility

**DOI:** 10.1038/s41598-018-19447-2

**Published:** 2018-01-18

**Authors:** Kristyna Sala, Andrea Raimondi, Diletta Tonoli, Carlo Tacchetti, Ivan de Curtis

**Affiliations:** 1 Cell Adhesion Unit - Division of Neuroscience, IRCSS San Raffaele Scientific Institute, 20132 Milano, Italy; 2 Experimental Imaging Center, IRCSS San Raffaele Scientific Institute, 20132 Milano, Italy; 3 San Raffaele Vita-Salute University, via Olgettina 58, 20132 Milano, Italy

## Abstract

Depletion of liprin-α1, ERC1 or LL5 scaffolds inhibits extracellular matrix degradation by invasive cells. These proteins co-accumulate near invadosomes in NIH-Src cells, identifying a novel invadosome–associated compartment distinct from the core and adhesion ring of invadosomes. Depletion of either protein perturbs the organization of invadosomes without influencing the recruitment of MT1-MMP metalloprotease. Liprin-α1 is not required for *de novo* formation of invadosomes after their disassembly by microtubules and Src inhibitors, while its depletion inhibits invadosome motility, thus affecting matrix degradation. Fluorescence recovery after photobleaching shows that the invadosome–associated compartment is dynamic, while correlative light immunoelectron microscopy identifies *bona fide* membrane–free invadosome–associated regions enriched in liprin-α1, which is virtually excluded from the invadosome core. The results indicate that liprin-α1, LL5 and ERC1 define a novel dynamic membrane-less compartment that regulates matrix degradation by affecting invadosome motility.

## Introduction

Different types of invasive cells including cancer cells, form specialized actin–rich membrane protrusions called invadopodia or podosomes, generally defined as invadosomes. These structures concentrate and secrete different types of proteolytic enzymes that are needed to locally degrade the extracellular matrix (ECM), in order to overcome the physical barriers met during invasive cell migration^1,2^. Invadosomes have a central actin-rich core decorated with metalloproteases that is surrounded by an adhesion ring consisting of adhesion and scaffold proteins like integrins, paxillin and vinculin^3^. Despite the important role of invadosomes during invasive cell migration, the molecular mechanisms driving their dynamic functional behaviour are not fully understood.

The scaffold and adaptor proteins liprin-α1, ERC1/ELKS and LL5 are part of functional plasma membrane associated networks that promote the turnover of integrin-mediated focal adhesions, and link the cell cortex and focal adhesions to microtubules^4–7^. The three proteins are important regulators of tumor cell migration and invasion *in vitro*^8–11^. Moreover liprin-α1 inhibits breast cancer cell invasion *in vivo*^12^, and is required for efficient ECM degradation by cancer cells, where it regulates the stability of invadopodia^10^. LL5β has been identified as a key component of synaptic podosomes, and is associated with the cytoplasmic side of the postsynaptic membrane of neuromuscular junctions, where it is required for proper acetylcholine receptor aggregation^13,14^.

Liprin-α1, ERC1/ELKS and LL5 proteins can interact with each other and colocalize at the front of migrating cells, where they promote adhesion turnover^5^. Moreover, these proteins are found at the cortex of non-motile cells where they are required to link the cell cortex to the microtubule network via CLASP proteins^4^. Time-lapse imaging has shown that during cell migration liprin-α1, ERC1 and LL5 define new highly polarized and dynamic cytoplasmic structures uniquely localized near the protruding cell edge^11^. Based on these findings we have proposed that these proteins assemble into plasma membrane-associated platforms, large membrane-less assemblies that promote protrusion at the cell front^15^. Membrane-less assemblies of proteins are implicated in several important cellular functions^16^. Here, we show that liprin-α1, ERC1 and LL5 promote the maturation and function of invadosomes, and that the three proteins colocalize near invadosomes, where they identify a novel dynamic, membrane–less invadosome–associated compartment.

## Results and Discussion

### Liprin-α1, ERC1 and LL5 promote ECM degradation by breast cancer cells and transformed fibroblasts

**Figure 1:**
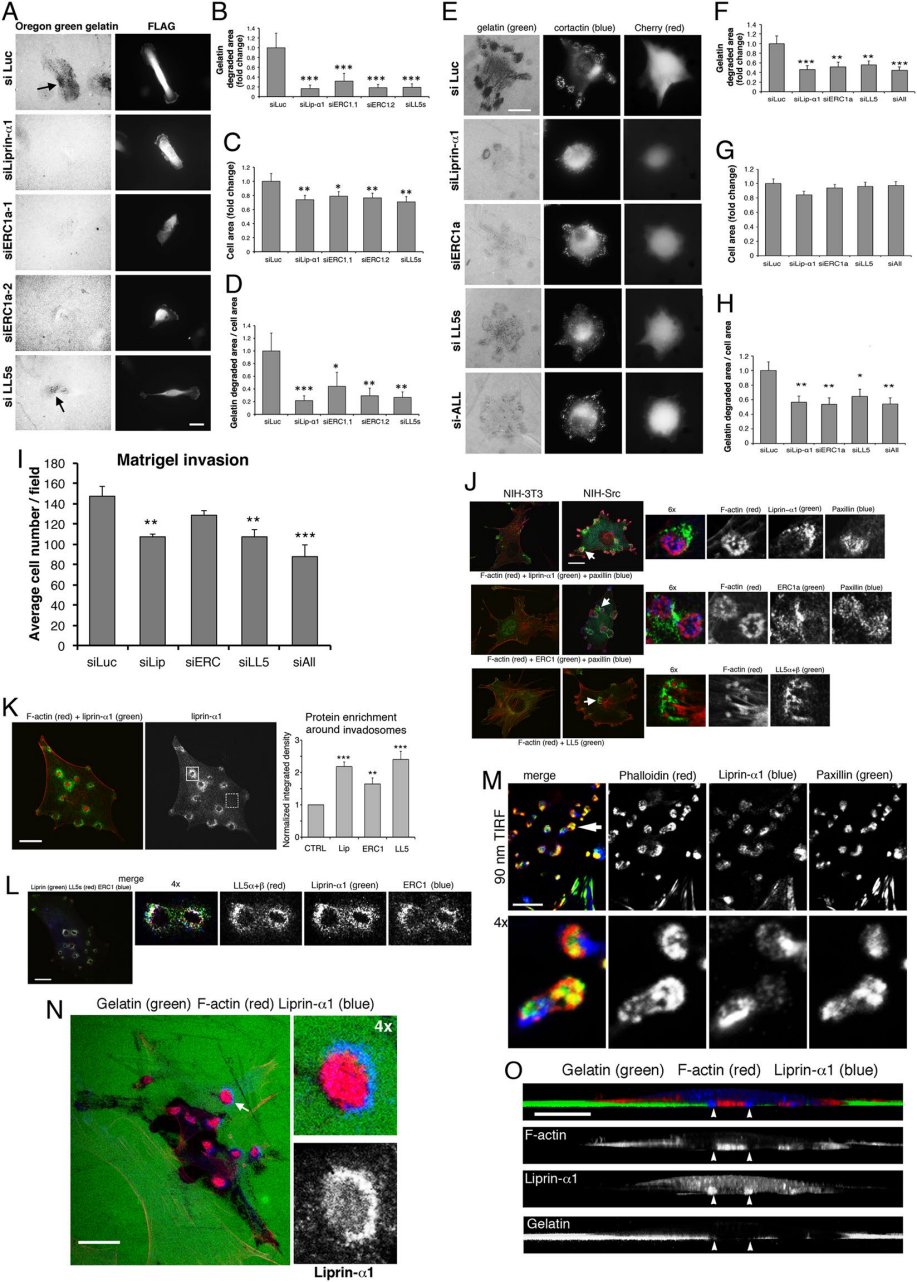
Liprin-α1, ERC1 and LL5 promote ECM degradation and identify a novel compartment associated to invadosomes. Inhibition of ECM degradation by MDA-MB-231 (**A**–**D**) or NIH-Src cells (**E**–**H**) after silencing liprin-α1, ERC1, LL5. Cells co-transfected with siRNAs and pFLAG-βGal/mCherry were replated on Oregon–green gelatin for either 5 h (**A**–**D**) or 1 h (**E**–**H**). Arrows in (**A**), dark areas of degradation. Bars, 20 µm. (**B,F**) Area of ECM degradation/cell, (**C,G**) projected cell area, (**D,H**) area of ECM degradation/projected cell area. Means ± s.e.m. [n = 25–50 cells (**B**–**D**); n ≥ 100 cells (**F**–**H**)]. ANOVA, Dunnet post hoc, *p < 0.05, **p < 0.01, ***p < 0.001 vs siLuc. (**I**) Matrigel invasion assay by cells transfected with control or specific siRNAs. Bars are means ± s.e.m. (n = 4–6 wells per experimental condition). ANOVA; Dunnet post hoc; **p < 0.01, ***p < 0.001 vs siLuc. (**J**–**K**) Cells fixed after 18 h on FN: endogenous liprin-α1, ERC1, LL5 accumulate at sites distinct from F-actin– and paxillin–positive regions of invadosomes (**J**). Enlarged areas in (**J**) are shown by arrows. (**K**) Left: liprin-α1 accumulation near invadosomes. Right: accumulation of endogenous proteins near invadosomes (e.g. full square, left image) compared to the diffuse cytoplasmic signal (dotted square, left image). Enrichments ± s.e.m. (n = 15–37 invadosomes, 7–8 cells); t-test: **p < 0.01, ***p < 0.001. (**L**) Endogenous liprin-α1, ERC1, LL5 colocalize near invadosomes. (**M**) TIRF: endogenous liprin-α1 is distinct from F-actin– and paxillin–positive regions of invadosomes. Enlargement (bottom) of the area shown by arrow (top). (**N**) Cells fixed after 18 h on Oregon–green gelatin, stained for endogenous liprin-α1 and F-actin. Enlargement (right): gelatin degradation by an invadosome surrounded by liprin-α1. The enlarged area is shown by the arrow. (**O**) Confocal 3D–reconstruction (Z axis, same cell of **N**): liprin-α1 (arrowheads) near F-actin–positive invadosomes with areas of ECM degradation. Bars, 20 µm (**J**–**N**), 10 µm (**O**).

Liprin-α1, ERC1 and LL5 cooperate to regulate cell motility and invasion by breast cancer cells^11^. MDA-MB-231 cells form functional invadopodia to degrade the ECM during invasion. 88% of MDA-MB-231 cells plated on Oregon–green gelatin showed ECM degradation, and silencing of either liprin-α1, ERC1 or LL5 protein in these cells (Supplementary Figure 1A; 77–93% protein decrease) strongly inhibited ECM degradation (Fig. 1A–D**)**. While knockdown of either protein reduced the percentage of ECM degrading cells, only liprin-α1 silencing slightly decreased the percentage of cells with invadopodia compared to controls (Supplementary Figure 1C). Rescue by expression of the human liprin–α1, which is resistant to silencing by the siRNA specific for murine liprin-α1, prevented the inhibition of ECM degradation by silencing of endogenous liprin-α1 (Supplementary Figure 1D).

Src activity is necessary for the formation and function invadosomes^17^. NIH-3T3 cells transformed by active Src-Y527F (NIH-Src cells) form large ECM–degrading invadosomes or podosomes^18^. Increased Src expression corresponds to strong Src activation detected by anti-Y416p-Src Ab (Supplementary Figure 2A). Virtually all NIH-Src cells with F-actin–positive dots or clusters (rosettes) of invadosomes showed active Src concentrated at these structures (Supplementary Figure 2B), and strong ECM degradation (Supplementary Figure 2C). Silencing of either liprin-α1, ERC1 or LL5 proteins, or their combined silencing in NIH-Src cells (85–90% protein decrease; 63–86% by triple silencing; Supplementary Figure 3A–C) dramatically inhibited ECM degradation (Fig. 1E–H), and reduced cell invasion *in vitro* (Fig. 1I**)**. Depletion of liprin-α1 decreased the percentage of cells with invadosomes and actively degrading invadosomes (Supplementary Figure 3D–E). These effects were not increased by triple silencing, suggesting that the three proteins cooperate to regulate the degradative efficiency of cells: depletion of either protein is sufficient to interfere with the functional complex. The results show that liprin-α1, ERC1 or LL5 proteins are important for ECM degradation by invasive breast cancer and transformed NIH-Src cells.

### Liprin-α1, ERC1 and LL5 define a novel compartment near invadosomes

Invadosomes in NIH-Src cells often form rosettes characterized by an F-actin–positive core, and a surrounding adhesive region or ring positive for focal adhesion proteins such as paxillin^19^. LL5β and ERC1/ELKS were described near podosomes in Src–transformed cells and myotubes during remodelling of the neuromuscular junctions^20^. Interestingly, we observed that liprin-α1, ERC1 and LL5 proteins strikingly co-accumulated near invadosomes of NIH-Src cells (Fig. 1J). Quantification of protein levels between areas near invadosomes and control invadosome-free areas confirmed that the three proteins were significantly enriched near invadosomes (Fig. 1K). Expression levels of the 3 proteins were not increased upon Src-induced transformation (Supplementary Figure 4). On the other hand neither protein evidently accumulated near invadopodia of MDA-MB-231 cells (Supplementary Figure 5A–C), where these proteins are found at the protrusive edge^11^. Also in cells plated on FN-coated Oregon–green gelatin the 3 proteins showed no particular accumulation near ECM degrading invadopodia (Supplementary Figure 5B–C). This may be due to differences in the structural organization of different types of invadosomes, with invadopodia representing incompletely organized ECM-degrading structures compared to invadosomes of NIH-Src or other cells^21^. In this direction, the accumulation of liprin-α1 near invadopodia has been linked to the presence of a paxillin–positive adhesion ring observed in different tumor cells^22^, but not in MDA-MB-231 cells (Supplementary Figure 5A).

Triple-immunostaining confirmed the co-accumulation of endogenous liprin-α1, ERC1 and LL5 near invadosomes of NIH-Src cells (Fig. 1L). Analysis by TIRF showed that they constitute a novel invadosome-associated compartment (IAC) near the ventral plasma membrane, which is distinct from the F-actin–positive core and from the associated paxillin–positive adhesion region/ring (Fig. 1M). Three-dimensional reconstructions of NIH-Src cells on Oregon–green gelatin confirmed the accumulation of endogenous liprin-α1 near actively degrading invadosomes, with the liprin-α1–positive compartment extending from the plasma membrane into the cytoplasm, at the sides of the protruding F-actin–positive core of ECM degrading invadosomes (Fig. 1N–O).

The IAC components ERC1, liprin-α1 and LL5 are required for efficient ECM degradation also by MDA-MB-231 cells, although a clear accumulation of these proteins as IACs near invadosomes could not be detected in MDA-MB-231 cells. This may be due to differences in the structural organization of different types of invadosomes. One hypothesis is that large morphologically identifiable IACs may only be detected at fully organized invadosomes, like the podosome-like invadosomes formed by NIH-Src cells. On the other hand, the same function may be carried on by smaller and possibly transient assemblies of the same IAC proteins that may remain morphologically undetectable near the less organized invadopodia-like invadosomes typical of MDA-MB-231 cells, which also lack an evident paxillin-positive adhesion ring. In this direction, it is interesting to observe that the overexpression of the constitutively active c-Src–Y527F mutant in MDA-MB-231 potentiated the formation of peripheral ECM-degrading invadosomes that were morphologically similar to those detected in NIH-Src cells: these invasodomes are surrounded by paxillin-positive adhesion rings and by IACs (Supplementary Figure 6), suggesting that the stable accumulation of liprin-α1, ERC1 and LL5 may depend on the presence of the adhesion-ring, and that Src activity may underlie this type of organization.

We have then looked for the presence of IACs near the invadosomes of other tumor cell types. In human epidermoid carcinoma A431 cells we observed liprin-α1–positive IACs near F-actin–positive invadosomes (Supplementary Figure 7A). Triple immunostaining showed that endogenous ERC1 and LL5 proteins often co-accumulated with endogenous liprin-α1, and that paxillin accumulated near invadosomes and IACs (Supplementary Figure 7B–C). In human fibrosarcoma HT1080 cells the situation was similar to that observed in MDA-MB-231 cells: HT1080 cells formed smaller invadopodia–like invadosomes, and the localization of liprin-α1 near F-actin-positive invadosomes was only seldomly observed. No clear accumulation of either IAC proteins or paxillin could be detected near the majority of invadosomes in HT1080 cells. On the other hand, as observed in MDA-MB-231 cells the three endogenous IAC proteins clearly co-accumulated at the cell edge near the lamellipodia of HT1080 cells (Supplementary Figure 7D–G).

### Liprin-α1, ERC1 and LL5 are necessary for invadosome development, but not for the recruitment of MT1-MMP at invadosomes

**Figure 2:**
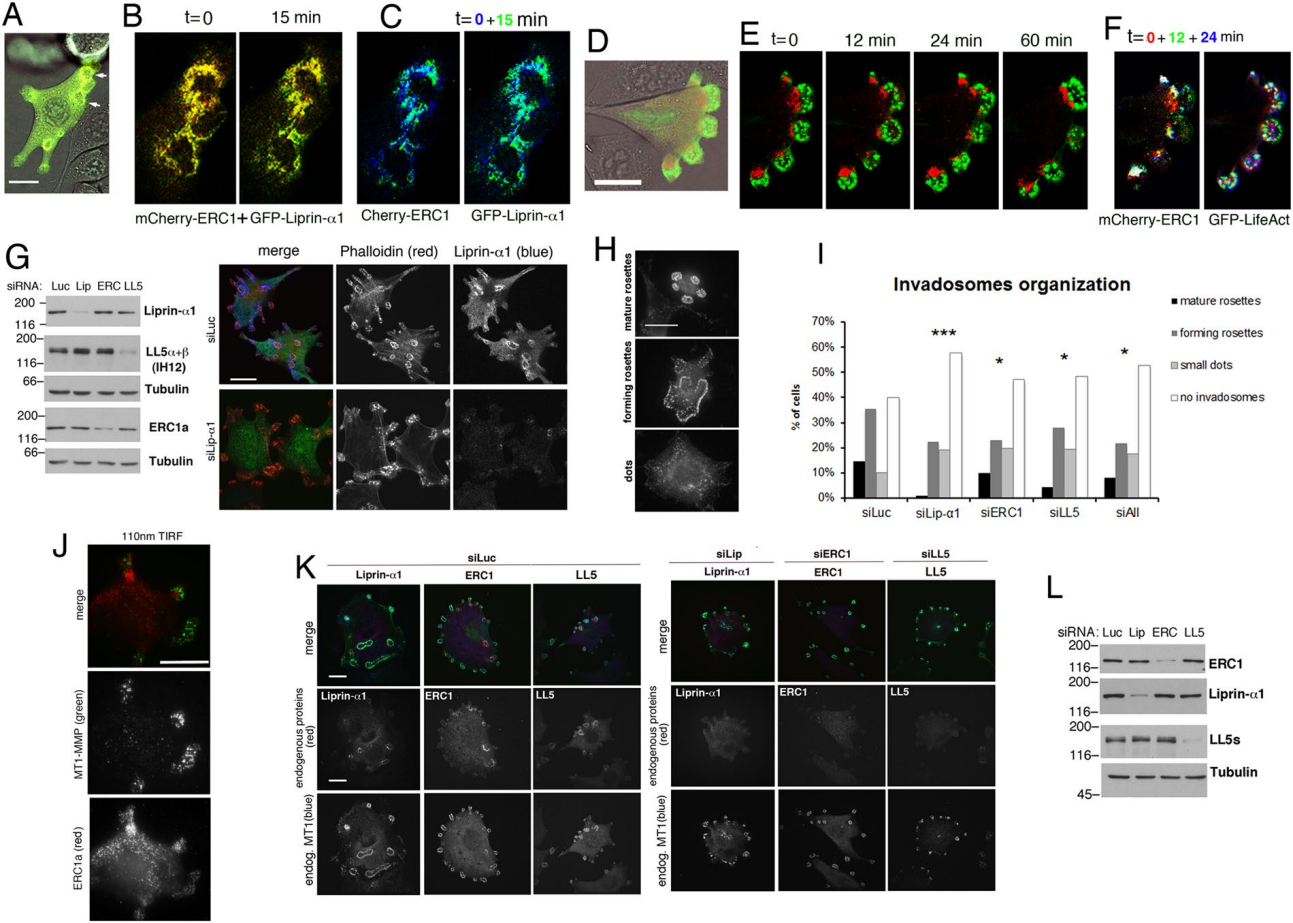
Liprin-α1, ERC1 and LL5 promote invadosome development. (**A**–**C**) Colocalization of GFP-Liprin-α1 and mCherry-ERC1 in live cells (**B**) is dynamic, as shown by color-coded time frames for either protein (**C**). (**B**–**C**) 2.5–fold enlargements of areas with arrows in (**A**). (**D**–**E**) No overlap of GFP-LifeAct (invadosome core) with mCherry-ERC1 (IAC) in live NIH-Src cells. Dynamic localization of both structures is shown by their color-coded distribution in time (**F**). (**G**–**L**) Silencing of IAC proteins perturbs invadosome organization. (**G**) Left: immunoblotting (30 µg protein/lane) from siRNA–transfected cells. Right: cells transfected with siRNAs, replated for 18 h before fixation. (**H**) Distinct types of invadosomes after 1 h on gelatin. Bar, 20 µm. (**I**) Relative abundance of different types of invadosomes in cells transfected with siRNAs, classified based on most organized invadosome structure present (rosettes, forming rosettes, dots, no invadosomes); n = 110–125 cells/condition; χ^2^ test vs siLuc: (siLip-α1 p = 0.00001, siERC1 p = 0.0389, siLL5 p = 0.0103. siAll p = 0.0133). (**J**) TIRF: distinct localization of endogenous MT1-MMP and ERC1 at the cells basal side. (**K**–**L**) Silencing of either liprin-α1, ERC1 or LL5 had no effect on endogenous MT1-MMP (cells replated 18 h). (**K**) Distribution of IAC proteins (red), MT1-MMP (blue) and F-actin (green) in cells transfected with siRNAs. (**L**) Immunoblotting on cells prepared as in (**K**).

Overexpressed liprin-α1, ERC1, and LL5β accumulated near invadosomes (Fig. 2A; Supplementary Figure 8) as the endogenous proteins (Fig. 1J). In living cells mCherry-ERC1 and GFP-Liprin-α1 largely colocalized near invadosomes, where they showed a dynamic behaviour (Fig. 2B,C; Supplementary movie 1). Time-lapse of cells cotransfected with mCherry-ERC1 and GFP-LifeAct to label the IAC and the F-actin–rich invadosomal core, respectively (Fig. 2D), revealed that both structures were dynamic, with continuous changes in their morphology, while maintaining a strict spatial relationship between them (Fig. 2E,F; Supplementary movie 2).

Depletion of either liprin-α1, ERC1 or LL5 (Fig. 2G; Supplementary Figures 9A–C; 10) only partially influenced the accumulation of the other proteins at IACs: ERC1 accumulation was decreased by depletion of either liprin-α1 or LL5, while accumulation of liprin-α1 and LL5 proteins were not inhibited by depletion of the other proteins (Supplementary Figure 11A). The percentage of cells with invadosomes18 h after plating was not decreased by knockdown of either protein (Supplementary Figure 11C). Depletion of either liprin-α1 or LL5 proteins affected the total invadosomal area/cell and the mean area of invadosomes, while silencing ERC1 had no such effects (Supplementary Figure 11D).

We evaluated the effects of protein depletion on the maturation of invadosomes at short times, in cells replated for 1 h on gelatin, by quantifying the percentage of cells with prevalence of either one of the three morphological categories of invadosomes corresponding to different phases of their maturation^23,24^: F-actin positive dots, clusters of dots (forming rosettes), mature rosettes (Fig. 2H). Depletion of either liprin-α1, ERC1 or LL5 as well as the combined depletion of the three proteins increased the percentage of cells without invadosomes, and decreased the percentage of cells with mature rosettes compared to control cells (Fig. 2I), indicating that each protein is required for the maturation of invadosomes.

The negative effects of protein silencing on ECM degradation (Fig. 1) may be due to a defect in the localization of metalloproteases at invadosomes^25–29^. The transmembrane metalloprotease MT1-MMP/MMP14 is a major player in ECM degradation by NIH-Src cells^27^. Its recruitment at invadosomes is an indication of their functional maturation^3^. MT1-MMP colocalized with the core of invadosomes in NIH-Src cells, while in parental NIH-3T3 cells had a diffuse signal (Supplementary Figure 12A). MT1-MMP was concentrated at ECM–degrading invadosomes of NIH-Src cells on Oregon–green gelatin (Supplementary Figure 12B). TIRF imaging showed that MT1-MMP localized distinctly from the ERC1-positive IACs on the basal side of cells (Fig. 2J). Depletion of either liprin-α1, ERC1 or LL5 resulted in loss of their accumulation near invadosomes, but did not affect MT1-MMP recruitment at invadosomes 18 h after plating cells (Fig. 2K–L; Supplementary Figure 10). To test for effects at earlier times during invadosome formation, we repeated the analysis with silenced cells replated for 1 h on gelatin. The recruitment of MT1-MMP at forming invadosomes was unaltered after efficient single/combined depletion of liprin-α1/ERC1/LL5 (Supplementary Figure 12C–E). Our results indicate that the three proteins are not required for the recruitment of MT1-MMP at invadosomes.

To look for possible effects of IAC perturbation on secreted metalloproteases (e.g. MMP9, MMP2) we performed gelatin zymography on lysates and media from cultures of cells depleted of liprin-α1. We found no detectable differences in the amounts of secreted metalloproteases in the medium from liprin-α1–depleted NIH-Src cells compared to control cells (Supplementary Figure 13).

### Liprin-α1 regulates invadosome motility

**Figure 3:**
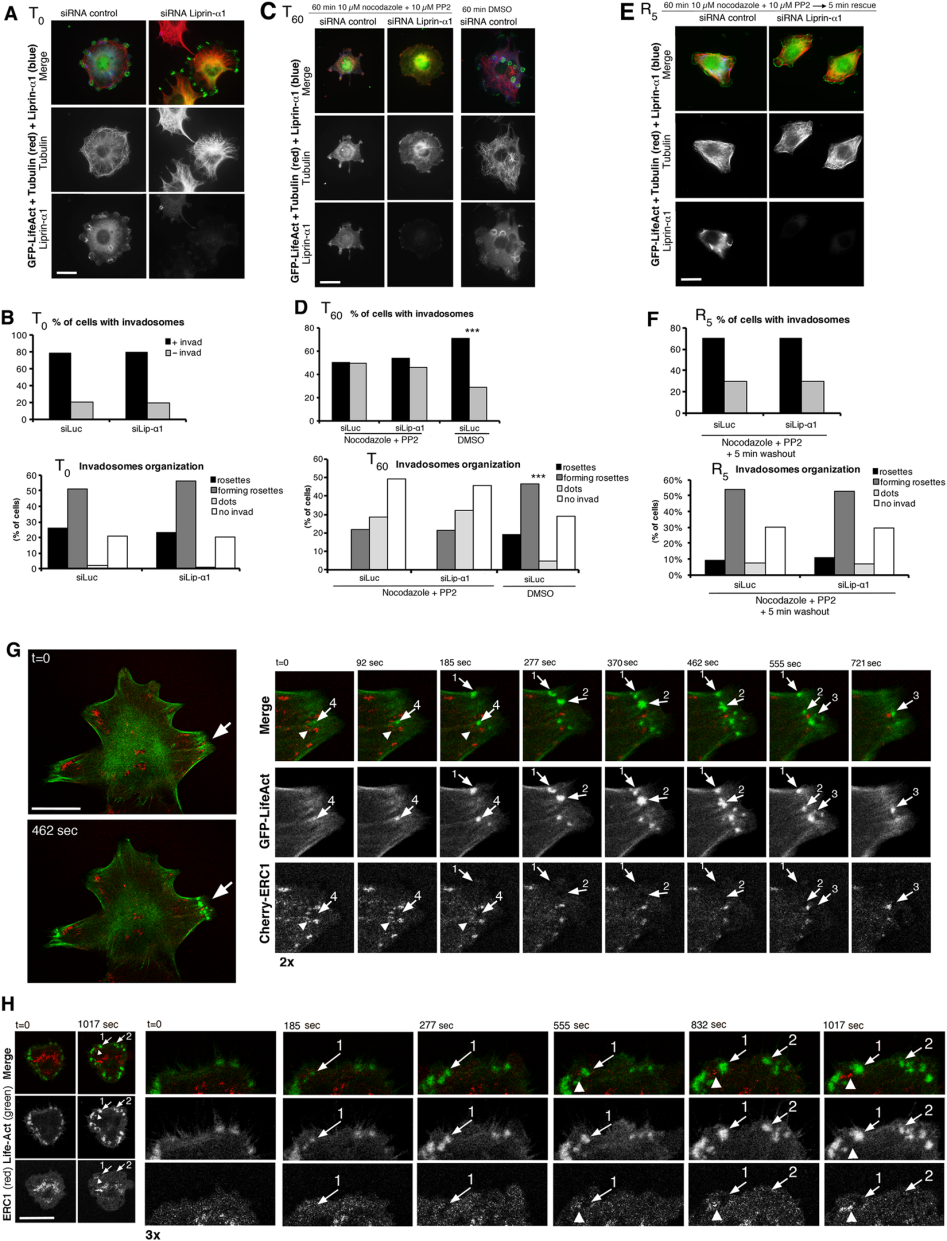
Liprin-α1 depletion does not affect invadosome formation, but affects their motility. (**A,C,E**) NIH-Src cells co-transfected with siRNAs and GFP-LifeAct (invadosomes), fixed before (**A**, T_0_) or after incubation with nocodazole and PP2 (**C**, T_60_), and after washout and incubation for 5 min to rescue invadosomes (**E**, R_5_). Immunostaining for GFP-LifeAct (green), tubulin (red), and liprin-α1 (blue). Bars, 20 µm. (**B,D,F**) Graphs: top, mean percentage of cells with/without invadosomes; bottom, invadosome organization: cells classified based on the highest organization of their invadosomes (n = 186–309). χ^2^ test: no differences with liprin-α1 and control siRNA; ***p < 0.001 for siLuc DMSO vs siLuc Noco + PP2 (**D**). (**G,H**) NIH-Src cells cotransfected with GFP-Life-Act and Cherry-ERC1 were incubated for 1 h with nocodazole and PP2. Images are frames from time-lapses recorded immediately after inhibitors washout. Arrows with numbers indicate different newly forming F-actin–positive invadosomes; arrowheads point to ERC1–positive areas near invadosomes. Bars, 20 µm.

To test if liprin-α1 is required for *de novo* formation of invadosomes, we treated NIH-Src cells with a combination of microtubules and Src kinase inhibitors, which are known to induce disassembly of invadosomes^19,30^. Microtubules were largely depolymerized after 30–60 min with 10 µM nocodazole, while the microtubule network was not perturbed by liprin-α1 silencing (Supplementary Figure 14A). On the other hand 1 h treatment with 10 µM PP2 inhibited Src activation as detected by immunoblotting with anti-Y416p-Src Ab^19^. Inhibition was reversed after washout of inhibitors and incubation for 5–10 min at 37 °C (Supplementary Figure 14B). Combination of 10 µM of each inhibitor for 1 h at 37 °C induced a significant disassembly of invadosomes (Supplementary Figure 16B). Increasing either the time of incubation or the concentration of inhibitors caused cell detachment (not shown). We thus analyzed the effects of liprin-α1 silencing on invadosomes formation following disassembly with inhibitors. The percentage of cells with high/low Src levels was not affected by treatments (Supplementary Figure 15). The effects were quantified as percentage of cells with invadosomes, and with prevalence of either one of the three morphological categories of invadosomes (rosettes, forming rosettes, dots, as in Fig. 2H–I). No differences were observed between liprin-α1 silenced and control cells before addition of inhibitors 18 h after plating (Fig. 3A–B; Supplementary Figure 16A). After 1 h with inhibitors, both control and liprin-α1–depleted cells were virtually depleted of mature rosettes, and had less forming rosettes and more cells with dots or no invadosomes compared to cells treated with DMSO only (Fig. 3C–D; Supplementary Figure 16B). Inhibition was reversible: inhibitors washout led to increased percentages of cells with invadosomes, including cells with forming or mature rosettes already after 5 min (Fig. 3E–F; Supplementary Figure 16C): again, no differences were observed in the capacity of recovery between liprin-α1–depleted and control cells, showing that liprin-α1 is not necessary for *de novo* formation of invadosomes.

We tested if during *de novo* formation of invadosomes, F-actin–positive cores appear before or after the formation of IACs. For this we performed time-lapse analysis on NIH-Src cells co-transfected with GFP-Life-Act and Cherry-ERC1 and treated with Src and microtubule inhibitors. Time-lapse analysis performed immediately after inhibitors washout showed that the appearance of F-actin–positive invadosomes occurred both in areas with, or devoided of pre-existing concentration of the IAC component Cherry-ERC1 (Fig. 3G–H). These results support the conclusion that IACs are not required for *de novo* formation of invadosomes, and indicate that detectable IACs associate to invadosomes after the appearance of the F-actin–positive cores (Fig. 3H).

**Figure 4:**
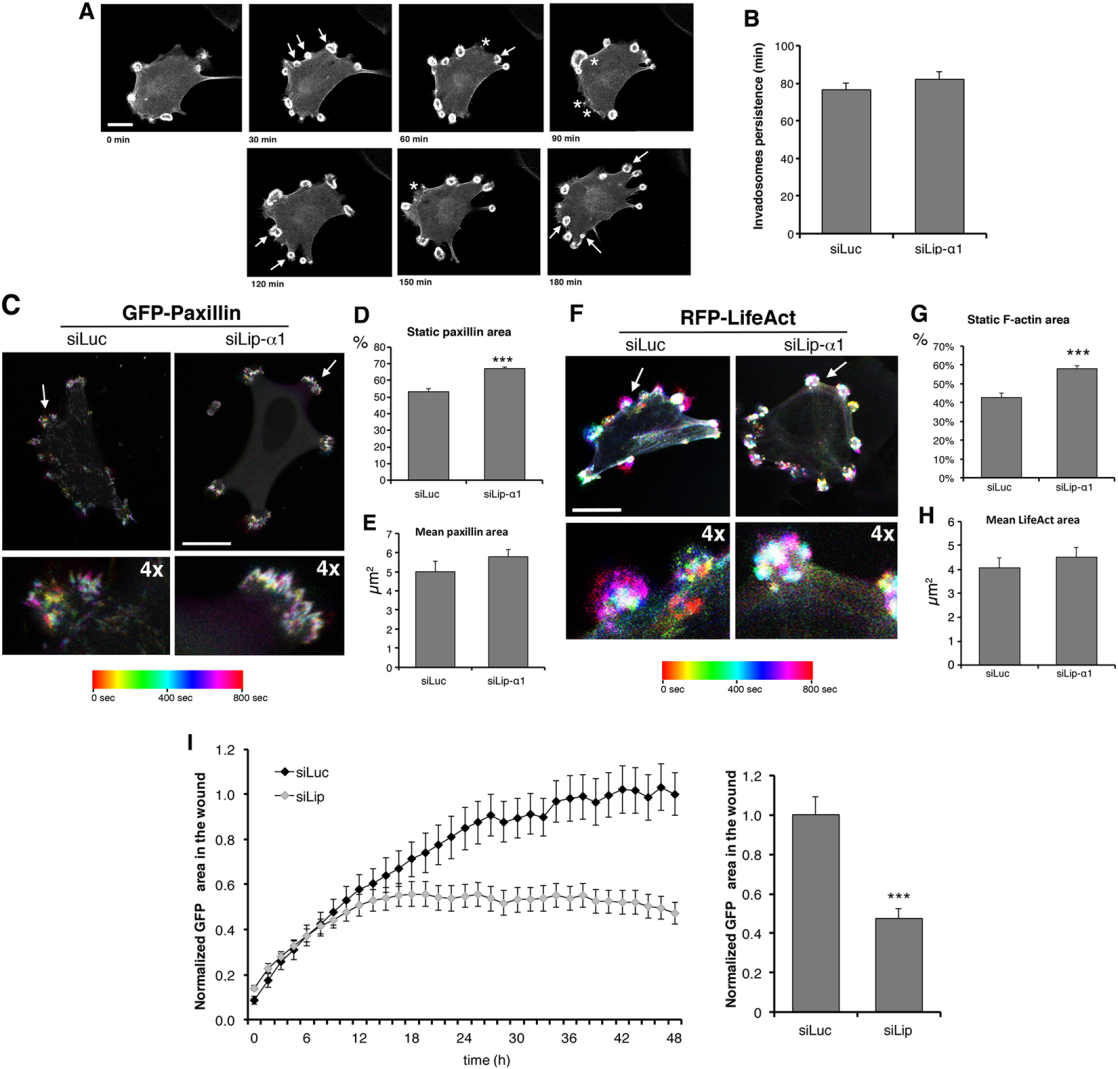
Liprin-α1 promotes invadosome motility. (**A**) Frames from time-lapse of cell co-transfected with control siRNA and GFP-LifeAct. Asterisks indicate disappearing invadosomes, arrows for new invadosomes. Bar, 20 µm. (**B**) Invadosome persistence was not affected by liprin-α1 silencing (n = 267–279 invadosomes, 13 cells). (**C**–**F**) Colour-coded maximal-intensity projection from time-lapse (20 frames, 40 sec intervals, ImageJ Time-lapse ColorCoder Plugin) of GFP-Paxillin–positive adhesion rings (**C**) and RFP-LifeAct–positive cores (**F**). Static rings/cores are white. (**D**–**E, G**–**H**) Mean ± s.e.m (n = 66–92 invadosomes, 6–8 cells, three experiments). (**D**) Percentage of static GFP-Paxillin–positive adhesion ring area (***p < 0.001, t-test); (**E**) GFP-Paxillin–positive adhesion rings area at T_n_ (*p* = 0.24, t-test). (**G**) Percentage of static RFP-LifeAct–positive invadosomal area (***p < 0.001, t test); (**H**) RFP-LifeAct–positive invadosome area at T_n_ (*p* = 0.45, t test). (**I**) Scratch-wound assay on monolayers of NIH-Src cells co-transfected with GFP and either control (siLuc) or liprin-α1 (siLip) siRNAs. Left: time course of migration into the wound. Right graph: means ± s.e.m. of the normalized area of GFP-positive cells into the wound area at 48 h. Values normalized to siLuc 48 h (n = 16 wells from 2 experiments; ***of the normalized area of*p* < 0.001, t-test).

We next tested the hypothesis that IAC proteins regulate invadosome motility, by comparing invadosome behaviour in NIH-Src cells co-transfected with either control or liprin-α1 siRNA together with GFP-Paxillin and RFP-LifeAct, to label adhesion rings and invadosomal cores, respectively (see Methods). Analysis by confocal live imaging (Fig. 4A; Supplementary movie 3) shows that liprin-α1 silencing did not affect invadosome persistence (Fig. 4B), but evidently inhibited the motility of invadosome–associated paxillin–positive adhesion rings (Fig. 4C–D) and LifeAct–positive invadosome cores (Fig. 4F–G), without influencing the total areas of adhesion rings and cores (Fig. 4E,H). Therefore, the movement of invadosomes is specifically inhibited by liprin-α1 silencing, suggesting that inhibition of ECM degradation upon liprin-α1 silencing may be due to the reduced motility of invadosomes. Reduced invadosomes motility may affect the migratory capacity of NIH-Src cells; accordingly, liprin-α1 silencing inhibited the migration of these cells (Fig. 4I), as already shown in other cell types^10,11^.

### A dynamic membrane-less compartment near invadosomes

**Figure 5:**
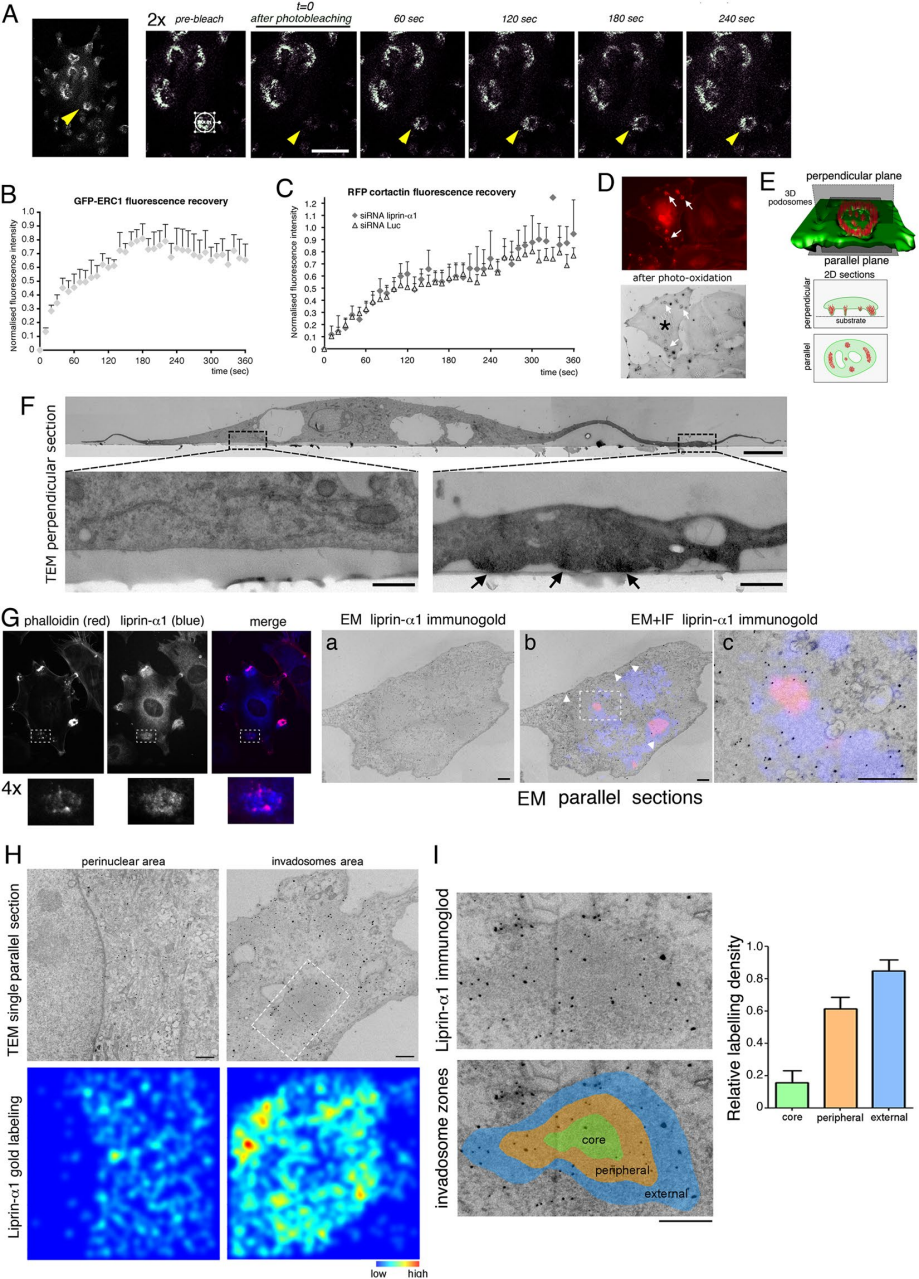
A dynamic membrane-less compartment near invadosomes. FRAP analysis. (**A**) Frames from time-lapse of a NIH-Src cell transfected with GFP-ERC1, to show the recovery of the fluorescent signal after photobleaching of a GFP-ERC1–positive IAC. Arrowheads indicate the photobleached region (encircled in the first enlarged panel, pre-bleaching). (**B**) Recovery of signal after photobleaching. Means ± s.e.m. (5 cells). (**C**) FRAP on RFP-cortactin–positive invadosomal cores from NIH-Src cells cotransfected with either control or liprin-α1 siRNA. No significant differences in the recovery of fluorescence by invadosomes from the cells of the two experimental conditions were observed (*p* > 0.05 by two-way ANOVA; n = 5–6 invadosomes/cells from 2–3 experiments). (**D**–**I**) CLEM analysis. (**D**) Cells on gelatin: TRITC-phalloidin–stained invadosomes (top, arrows) are dark by phase contrast after photoconversion (bottom). (**E**) Sections for TEM, parallel or perpendicular to the substrate. (**F**) TEM micrographs (section perpendicular to the substrate) of photoconverted cell (asterisk in **D**). Boxes: area of cytoplasm without (left) or with invadosomes (right, electron dense after photoconversion of labelled F-actin). (**G**) Cells labelled for endogenous F-actin and liprin-α1 (immunofluorescence on the left; below are shown 4-fold enlargements of boxed invadosomes), were incubated with nanogold–conjugated anti-rabbit Ig for immuno-EM. The three panels on the right are EM micrographs of the boxed invadosomal area shown on the left: (**a**) immunogold–labelled endogenous liprin-α1 in the invadosomal area; (**b**) overlay of F-actin (fluorescent) with liprin-α1 (immuno-EM); (**c**) enlargement showing immunogold–labelled endogenous liprin-α1 concentrated around the colored F-actin core (from image boxed in **(b**)). Liprin-α1 is almost absent at the invadosome core. (**H**) Sections from stacks to quantify the distribution of liprin-α1 in invadosomal (right) and non-invadosomal areas (left). Bottom: color-coded distribution of immunogold particles in overlaid stacks of 5 sections in correspondence of the same area shown in the respective top panels (liprin-α1). (**I**) Distribution of gold particles near an electron dense F-actin–rich invadosomal area (boxed in **H**). Lower panel: colors represent centre, periphery, and adjacent area of electron dense invadosomal core. Graph: means ± s.e.m. of gold particles density. Scale bars: 5 µm (**F**, upper panel); 0.5 µm (F, lower panels); 1 µm (**H**,**I**).

The dynamic behaviour of IACs was supported by fluorescence recovery after photobleaching (FRAP) on cells expressing GFP-ERC1 (Fig. 5A). GFP-ERC1 fluorescence in photobleached IACs recovered quite rapidly (t_1/2_ = 40 sec) to about 70% of the levels of fluorescence before bleaching (Fig. 5B), suggesting that IACs dynamic behavior is accompanied by a rapid turnover of a large fraction of their molecular components by exchange with the surrounding cytoplasm.

We checked if altering the IACs by depletion of liprin-α1 affected the dynamics of fluorescence recovery within the invadosomal core. For this we cotransfected NIH-Src cells with either control or liprin-α1 siRNAs together with RFP-cortactin as a marker of invadosomal cores. FRAP analysis showed that depletion of liprin-α1 had no detectable effect on the recovery of fluorescent cortactin in invadosomes compared to control cells (Fig. 5C). These data suggest that perturbation of IACs did not affect the exchange rate of intrinsic components of the invadosomal core.

To gain a better insight of the liprin-α1–positive compartment, we performed ultrastructural analysis on NIH-Src cells plated on gelatin. We used Correlative Light Electron Microscopy (CLEM) to identify *bona fide* invadosomes and invadosome–associated regions, based on their localization by fluorescence confocal microscopy. Photooxidation of TRITC-phalloidin labelled the F-actin–rich areas corresponding to invadosomes, forming electron dense precipitates visible by transmission electron microscopy (TEM) (Fig. 5D). Cells on gelatin were used to prepare thin sections for TEM with a cutting plane either parallel or perpendicular to the substrate (Fig. 5E). TEM revealed electron dense F-actin–rich areas corresponding to the same invadosomes observed by immunofluorescence (Fig. 5F). We next used cells double labelled with fluorescence for F-actin and endogenous liprin-α1 without photoconversion, to avoid loss of information due to interference by the dark precipitates. We could identify regions corresponding to invadosomes on thin sections parallel to the plane of the substrate: invadosomes included cytoplasmic areas with homogeneous electron density, possibly due to concentrated actin filaments (Supplementary Figure 17). To look at the relationship between the F-actin–rich invadosome core and the nearby liprin-α1–enriched region, we performed CLEM on cells double labelled with fluorescent phalloidin for F-actin, and nanogold–conjugated anti-rabbit Ig to recognize Ab-labelled endogenous liprin-α1 (Fig. 5G). We could localize invadosomes as regions positive for TRITC-phalloidin, and the nearby liprin-α1–positive areas (Fig. 5G). Endogenous liprin-α1 was preferentially at the periphery of invadosomes, with poor liprin-α1 signal found in F-actin–positive cores. Interestingly, the localization of the immuno-gold complexes was not associated to any morphologically identifiable intracellular membrane-bound compartment, while some gold particles localized at the cytoplasmic face of the plasma membrane, as expected (Fig. 5H, arrowheads). Quantitative analysis confirmed that labelling for endogenous liprin-α1 was specifically concentrated around amorphous regions corresponding to the invadosomal F-actin–rich patches (Fig. 5I). These results provide evidence for the accumulatation of endogenous liprin-α1 into a membrane-less compartment closely associated to invadosomes.

## Conclusions

In this study we have shown that the scaffold proteins liprin-α1, ERC1 and LL5 co-accumulate at IACs, a novel dynamic compartment associated to invadosomes. We show that the three proteins promote the morphological and functional maturation of invadosomes (Fig. 1), but are dispensable for their formation. IACs are involved in the matrix degradation process occurring during invasion. In support of the role of IACs as functional regulators of ECM–degrading invadosomes, the morphological characterization of IACs by confocal and TIRF imaging has shown that these structures are highly dynamic, and remain closely associated to invadosomes (Fig. 2). Interestingly, FRAP analysis shows a rapid turnover of the molecular components of IACs that exchange with molecules in the cytoplasm. This finding is consistent with the hypothesis that IACs may derive from the association of protein components into membrane–less organelles^15,31,32^. Support to this hypothesis has been provided here by the use of CLEM (Fig. 5), which has allowed us to show that the IAC component liprin-α1 is concentrated in regions around F-actin-enriched invadosomes that are not associated to any morphologically identifiable intracellular membrane-bound compartment.

One possibility is that IAC proteins influence invadosomes by regulating the adhesive regions near invadosomes, which have been proposed to be crucial for invadosome maturation and function rather than for their formation^33^. This may explain the apparently contradictory results showing that depletion of liprin-α1 does not influence the *de novo* formation of invadosomes after their disassembly by inhibitors (Fig. 3), whereas depletion of liprin-α1, ERC1 and LL5 inhibits the capacity of cells to degrade the ECM. In this context, the IAC component liprin-α1 promotes the motility of invadosomes and invadosome–associated adhesions (Fig. 4): the reduced motility of invadosomes upon liprin-α1 silencing may reduce their degradative capacity in time, resulting in overall reduced ECM degradation, even if the localization of the membrane–bound metalloproteases and the secretion of soluble metalloproteases remains unaffected. Based on these observations, we propose the hypothesis that the observed reduction of ECM degradation in IAC protein–silenced cells is not due to a direct effect on the metalloproteases, but to the reduced motility of the invadosomes. One possibility is that the reduction of invadosome motility by liprin-depletion may restrict the lytic action of MT1-MMP on ECM, thus reducing the efficacy of ECM degradation. As a consequence, this may also explain the negative effects of silencing the IAC proteins on cell motility and invasion.

Given the implication of liprin-α1 and associated proteins in breast cancer cell invasion^10,12,22^, further understanding of the molecular mechanisms driving the assembly and function of IACs appears to be highly relevant to the understanding of the mechanisms that drive the formation of metastasis.

## Materials and Methods

### Antibodies and reagents

Antibodies and reagents used in this study include: anti-α-tubulin monoclonal antibody (mAb) and polyclonal antibodies (pAbs) against FLAG and LL5*β* (Sigma-Aldrich St. Louis, MO); mAb anti-paxillin (cl. 349); pAb anti-paxillin and mAb anti anti-liprin-α1 (Santa Cruz Biotechnology, Santa Cruz, CA); pAb anti-GFP (Life Technologies, Paisley, Scotland, UK); affinity purified anti-liprin-α1 pAb^9^; pAb anti-liprin-α1 (Protein Tech, Chicago, IL); mAb anti-ERC1a (clone ELKS-30), pAb anti MMP14 (MT1-MMP) and chicken pAb anti-GFP (Abcam, Cambridge, UK); mAb anti-cortactin cl. 4F11 (Millipore, Billerica, MA); pAb anti-phospho Src (Tyr416) (Cell Signalling Technology); mAb anti-LL5αα and *β* (cl. IH12) was a gift from J. Sanes^13^; hamster mAb anti-LL5α was a gift from Y. Mimori-Kiyosue^34^; mAb anti Src cl. 327 was a gift from S. Courtneidge^35^ Alexa-488, Alexa-568 and Alexa-647 secondary Abs and Oregon 488-gelatin (Life Technologies, Paisley, Scotland, UK); FITC- and TRITC-conjugated phalloidin (Sigma-Aldrich, St. Louis, MO); goat-anti rabbit nanogold–conjugated secondary Ab (Nanoprobes, Stony Brook, New York), fibronectin (BD Biosciences, San Jose, CA); peroxidase-conjugated anti-rabbit and anti-mouse secondary Abs, and Enhanced Chemiluminescence (ECL) Detection System (Amersham Biosciences, Little Chalfont, UK); Peroxidase-conjugated anti-hamster secondary Ab (Life Technologies); nocodazole and poly-L-lysine hydrobromid (Sigma-Aldrich, St. Louis, MO); PP2 Src kinase inhibitor (Calbiochem) and G418 (Merck, Darmstadt, Germany).

### Plasmids and siRNAs

The following plasmids were used: pFLAG-Liprin-α1 and pEGFP-Liprin-α1 (human liprin-α1) and FLAG-*β*Gal^36^; pEGFP-C1, m-Cherry-C1, and pRFP (Clontech Laboratories, MountainView, CA); pEGFP-ERC1a (murine ERC1a) from Y. Takai^29^; pEGFP-LL5*β* (murine LL5*β*) from J. Sanes^13^; pEGFP-LifeAct^37^; mCherry-ERC1a^5^. RFP-LifeAct was obtained from pEGFP-LifeAct by cloning into pRFP vector. GFP-paxillin was a kind gift from dr. Victor Small (Austrian Academy of Sciences, Vienna, Austria). The plasmids pSGT-Y527F-Src^38^ and RFP-cortactin^39^ were as described.

SiRNAs were from Qiagen (Hilden, Germany) and Life Technologies. SiRNAs for human proteins were as published^4,5,9,34^, and targeted the following sequences: liprin-α1 CCAAGGTACAAACTCTTAA; ERC1a: GTGGGAAAACCCTTTCAAT and CCAACAGTACGGGAGGGAG; LL5*β*: GGAGATTTTGGATCATCTA and GGATCTACCTCATAGCGTA. LL5α: CCATCAGCCTGAGTGAATA; control siRNA for luciferase: CATCACGTACGCGGAATAC. SiRNAs for mouse proteins targeted the following sequences: liprin-α1: GCTGGATGCTATCAACAAA and ERC1a: GAAGGAAGTATTAAGAGAA were as described^40^; LL5*β*: AGAGAAGAACAATCTAATA.

### Cell culture and transfection

MDA-MB-231 human breast adenocarcinoma cells were grown in DMEM/F12 1:1 with 10% FBS (Euroclone, Wetherby, UK). NIH 3T3 mouse embryonal fibrobasts stably transformed with the retroviral constitutively active Y527F c-Src mutant (NIH-Src, a kind gift from S. Corallino, IFOM, Milan) were cultured in DMEM with 10% FBS and maintained under selection with 0.75 mg/ml G418 (Merck, Darmstadt, Germany). A431 cells were cultured in DMEM with 10% FBS. HT1080 were cultured in EMEM with 10% FBS.

Cells were transfected with Lipofectamine-2000® (Life Technologies). For 6-well plates 50–100 nM siRNA and/or 1–6 μg plasmid DNA in Optimem® transfection medium were used for each transfection. After 4–6 h at 37 °C the transfection medium was replaced with complete medium, and cells processed after 24–48 h later.

### Immunochemical analysis

Cells cooled on ice were washed twice with of ice-cold TBS (150 mM NaCl, 20 mM Tris-HCl pH 7.5), and solubilised with 50–150 *μ*l of lysis buffer (0.5% Triton X-100, 150 mM NaCl, 20 mM Tris-Cl pH 7.5, 2 mM MgCl_2_, 1 mM NaV, 10 mM NaF, and anti-proteases 1× Complete® (Roche, Manheim, Germany). Lysates were rotated for 15 min at 4 °C, insoluble material removed by centrifugation at 12000 g at 4 °C for 10 min. Protein determination was done using Bradford protein assay reagent (Bio-Rad, Hercules, CA). Lysates were loaded for SDS-PAGE. Proteins were transferred to 0.45 μm PROTRAN® nitrocellulose membranes (GE Healthcare Amersham Biosciences, Little Chalfont, UK), which were incubated with primary antibodies, horseradish peroxidase-conjugated secondary antibodies, and revealed by ECL (GE Healthcare Amersham Biosciences, Little Chalfont, UK). Quantification of protein levels was done by densitometric analysis on scanned films using ImageJ. Values of specific protein bands were normalized to the levels of internal protein standards in the lysate (α-tubulin or calnexin). Some filters were incubated for 5–10 min at room T with mild stripping buffer (0.2 M glycine, 0.1% SDS, 1% Tween-20, pH 2.2) or for 30 min at 57 °C with harsh stripping buffer (62.5 mM Tris-HCl pH 6.8, 2% SDS, 100 mM 2-mercaptoethanol) washed at neutral pH, and then reprobed for immunoblotting with different antibodies.

### Gelatin degradation

Gelatin degradation was detected as published^25^. Glass coverslips coated for 1 h at room T with 0.5 mg/ml poly-L-lysine (Sigma-Aldrich, St. Louis, MO) were quenched 15 min at 4 °C with 0.5% glutaraldehyde in PBS, and then coated for 10 min at room T with Oregon–green–conjugated gelatin (Life Technologies) diluted 1:6 in 0.2% gelatin in PBS. Subsequently the coverslips were either additionally coated with 10 µg/ml FN in PBS for 1 h at 37 °C (for MDA-MB-231 cells), or treated with 1% NaBH_4_ in PBS for 15 min (for NIH-Src cells). Cells were plated on gelatin-coated coverslips for either 5 h (MDA-MB-231), or for 1 h (NIH-Src) before fixation and immunostaining. Gelatin degradation was detected at a Zeiss Axiovert 135 TV microscope equipped with Hamamatsu CCD Orca II camera and Plan-Apochromat 63× (NA 1.4) lens. The dark areas of gelatin degradation and the projected cell areas were quantified by ImageJ on thresholded images. Invadopodia were identified by immunostaining for cortactin. Data were pooled from 2-3 independent experiments.

To quantify the localization of MT1-MMP at invadosomes, control and transfected NIH-Src cells were plated for 1 h on non-fluorescent gelatin-coated coverslips. After fixation cells were stained for invadosomal marker (phalloidin) and MT1-MMP. Confocal images were analyzed for the localization of MT1-MMP at invadosomes. Both the total area of invadosomes/cell and the integrated density of MT1-MMP signal at invadosomes were measured. The ratio between MT1-MMP integrated density and the total invadosome area represents the mean density of MT1-MMP signal at invadosomes.

### Matrigel invasion assay

NIH-Src cells transfected for 48 h with the indicated siRNAs were seeded on Matrigel–coated transwells (Matrigel, BD Transduction, San Jose, CA; transwells 0.8 µm pores, Millipore, Billerica, MA) in DMEM 0.1%BSA (20000/100 µl/ transwell) with lower chambers filled with complete medium. After 24 h, cells were fixed with MetOH and coloured with Crystal Violet. Invading cells at the bottom of the transwell membrane were counted (n = 4–6 transwells per experimental condition, from 2–3 experiments).

### Scratch-wound assay

NIH-Src cells co-transfected with GFP and indicated siRNAs were replated after 24 h in 96 well plate (20,000 cells in 100 µl of complete medium per well (96-well ImageLock Plate, Essen BioScience, Ann Arbor, MI), and incubated for 2.5 h to allow cell attachment. Wounds 700 µm wide were created with a WoundMaker Tool (Essen BioScience), cells were washed with PBS, supplied with complete medium and imaged every 1.5 h for 48 h with IncuCyte Live-Cell Imaging System equipped with 10× lens (Essen BioScience). The percent of wound area occupied by the GFP-positive cells was calculated at each time point with the IncuCyte Scratch Wound Assays Software (Essen BioScience).

### Morphological analysis

Cells grown on uncoated or FN-coated glass coverslips were washed and fixed for 12 min at room T in 3% paraformaldehyde in PBS+ (Ca^2+^, Mg^2+^). After washing and quenching for 10 min with 50 mM NH_4_Cl in PBS+, cells were permeabilized for 4 min with 0.1% Triton X-100 or saponin 0,1%. Coverslips were incubated for 2 h at room T with primary antibodies, then for 45 min with secondary antibodies, and finally mounted in ProLong® Gold Antifade Reagent (Life Technologies). For z-stacks imaging, coverslips were mounted in glycerol 70% with 6,5 mM p-phenylenediamine (Sigma-Aldrich, St. Louis, MO) in PBS and sealed with transparent nail polish.

Wide field images were acquired with Zeiss Axiovert 135 TV with QImaging Exi-Blue equipped with a Hamamatsu CCD Orca II camera, with Plan-APOCHROMAT 63× (NA 1.4) lens. Confocal images were acquired at a Perkin Elmer UltraVIEW spinning disk confocal microscope equipped with EM-CCD camera, with Plan-Apochromat 63× (NA 1.4) lens; alternatively images were taken at a Leica TCS SP8 SMD FLIM laser scanning confocal microscope equipped with HC PL APO CS2 63× (NA 1.4) lens. Total internal reflection fluorescence (TIRF) images were acquired at a Leica SR GSD 3D TIRF microscope equipped with EM-CCD and CMOS camera and HCX PL APO 63× (NA 1.47) lens or HC PL APO 160× lens (NA 1.43).

Quantification of invadosomes. NIH-Src cells co-transfected with p-EGFP-C1 and siRNAs were replated on glass coverslips and cultured for 18 h before fixation and staining with phalloidin, and immuno-staining for the detection of invadosome-associated proteins. The number and area of invadosomes were evaluated by analysis with public domain NIH Image software ImageJ. The total area of invadosomes per cell was measured after applying a fixed threshold to identify the invadosomes, and subsequently invadosomes were counted manually. Projected cell area was measured applying threshold on GFP channel.

Quantification of protein enrichment around invadosomes. Thresholded confocal images from transfected NIH-Src cells co-stained for a invadosomal marker (phalloidin) and for components of the liprin-α1/ERC1a/LL5 complex were used to evaluate the accumulation of these proteins near central invadosomes. The selected areas were defined by applying a 10 pixels extension around the invadosomal area; the integrated density of signal for liprin-α1, ERC1a, or LL5 was then measured in this extended invadosomal area. Subsequently the integrated density of the signal for each protein was measured in a randomly selected equivalent control invadosome-free area. Enrichment of each protein was calculated as the ratio between the signal near invadosomes and the signal in control areas. Data were pooled from 2 independent experiments.

For *de novo* formation of invadosomes, NIH-Src cells co-transfected for 48 h with siRNAs and GFP-LifeAct and replated on 13 mm glass coverslips (12,500 cells per coverslip), were treated with inhibitors 18 h after seeding. Cells were incubated for the indicated times at 37 °C with 10 µM nocodazole and 10 µM PP2 Src inhibitor in DMSO, or with DMSO alone as control. Invadosome formation was evaluated by removing the inhibitors, washing cells twice with PBS, and incubating them for 5–10 min at 37 °C in culture medium. Cells fixed with either 3% paraformaldehyde or with Methanol at −20 °C to preserve microtubules, were analyzed to evaluate the presence and organization of invadosomes in GFP-LifeAct–positive cells (rosettes, forming rosettes, dots). Src and pSrc were detected by immunostaining and by immunoblotting with anti-Src and anti–Y416pSrc Abs. About 300 cells from three independent experiments were analysed. Efficacy of Src inhibition by PP2 was confirmed by the decrease of invadosomes, as well as by immunoblotting with anti-Src and anti–phospho-Src Abs. Depolymerization of microtubules by nocodazole was confirmed by immunostaining with anti-tubulin Abs.

### Live-cell imaging

For live-cell imaging NIH-Src cells co-transfected with siRNA and plasmids were replated (40,000 cells per dish) on 3.5 cm diameter glass-bottom MatTek dishes (MatTek Corporation, Ashland, MA) and cultured for 18 h. Before imaging culture media was replaced with imaging media (without phenol red). Cells were imaged at a confocal microscope Leica TCS SP8 SMD FLIM equipped with HC PL APO CS2 63× (NA 1.4) lens, adaptive focus control and Oko-Lab stage incubator (T, CO_2_) with use of LasX software (Leica) at the same focal plane. For each time-lapse experiment using siRNA, a control coverslip was fixed before imaging to verify protein silencing by immunofluorescence. Cells in mitosis or going out of focus during acquisition were excluded from the analysis.

Isolated cells were analyzed for GFP-LifeAct–positive invadosomes persistence, evaluated as the time each invadosome was detectable over a 3 h period (one frame every 3 min). Invadosomes were manually tracked from first to last frame of its persistence. In case of invadosomes fusion the bigger one counted as persisting while the smaller as disappeared, in case of invadosome split the bigger counted as persisting from the original invadosome while the smaller counted as a new one.

For the analysis of invadosome motility, cells co-transfected with GFP-Paxillin and RFP-LifeAct were imaged at a Leica TCS SP8 confocal microscope with 63× lens. For each sample, 20 frames were taken at 40 sec intervals. To evaluate the dynamics of invadosomes, a macro was created by ImageJ for automatic analysis of the area of either GFP-paxillin–positive adhesion rings or RFP-LifeAct–positive invadosomes. Cells with similar invadosome organization (forming rosettes) were chosen for acquisition. Time-lapses were processed with ImageJ StackReg Plugin for analysis. For each experiment, a control coverslip was fixed before imaging to verify silencing of liprin-α1 by immunofluorescence.

The motility of invadosomes/adhesion rings were evaluated as the percentage of area shared between pairs of two consecutive time points T_n_ and T_n+1_ (n = 0–20), calculated as follows: the GFP-Paxillin–positive (or RFP-LifeAct–positive) area overlapping between T_n_ and T_n+1_ was divided by the area at T_n_, and expressed as the percentage of static invadosomal area. This measure was repeated for consecutive pairs of time points up to T_20_; the mean of the 20 values obtained for each invadopodium was expressed as percentage, and defined as *static GFP-Paxillin area* for adhesion rings, or *static RFP-LifeAct area* for invadosome cores. Measurements were made on 66–92 invadosomes from 6–8 cells per experimental conditions, from 3 independent experiments.

For *de novo* formation of invadosomes, 40,000 NIH-Src cells co-transfected with GFP-Life-Act and Cherry-ERC1 were replated in 3.5 cm diameter glass-bottom MatTek dishes (MatTek Corporation, Ashland, MA), and cultured for 18 h. Before imaging cells were incubated for 1 h at 37 °C with 10 µM nocodazole and 10 µM PP2 Src inhibitor in complete medium. Cells were washed once with PBS, supplied with complete medium for imaging (without phenol red), and immaged immediately (one frame every 18.5 sec). Cells were imaged at a confocal microscope Leica TCS SP8 SMD FLIM equipped with HC PL APO CS2 63× (NA 1.4) lens.

### FRAP analysis

For FRAP analysis, cells were imaged at a confocal microscope Leica TCS SP8 SMD FLIM equipped with HC PL APO CS2 63× (NA 1.4) lens, adaptive focus control and Oko-Lab stage incubator (T, CO_2_), and FRAP module with LasX software (Leica). For GFP-ERC1 analysis NIH-Src cells plated on 3.5 cm diameter glass-bottom MatTek dishes (MatTek Corporation, Ashland, MA) were cotransfected with GFP-ERC1a and LifeAct-RFP for 24 h. A 10 µm diameter circular region of interest (ROI) was bleached at 35% laser power at 488 nm with 2 iterations every 2.5 sec. After bleaching, images were taken within the same focal plane every 10 sec for 10 min to monitor fluorescence recovery. For RFP-cortactin analysis NIH-Src cells were cotransfected with RFP-cortactin and the indicated siRNAs for 24 h, replated on 3.5 cm diameter glass-bottom MatTek dishes and imaged after 24 h. An 11 µm diameter circular region of interest (ROI) was bleached at 45% laser power at 568 nm with 4 iterations every 2.58 sec. After bleaching, images were taken within the same focal plane every 10 sec for 20 min to monitor fluorescence recovery. The recovery of the GFP/RFP signal was measured using ImageJ by calculating the fluorescent intensity at each time point as follows: R = [F(t)-F(0)]/[F(pre-bleach)-F(0)], where F(t) is the intensity of fluorescence at time t: F(t) = [F(ROI(t))-F(bcg(t))]/[F(ctrl(t))-F(bcg(t))], where “bcg” stands for background fluorescence outside the cell, and “ctrl” stands for control area in the cytosol^41,42^. T_1/2_ was identified as the first time point that achieved half of the intensity of the plateau (beginning of plateau defined manually). The immobile fraction was defined as the difference between the value of fluorescence before bleaching and the value of fluorescemce at plateau. Data are presented as means ± s.e.m.

### Correlative Light Electron Microscopy (CLEM)

For DAB (3,3′-diaminobenzidine tetrahydrochloride) photo-oxidation cells plated on gelatin–coated coverslips were fixed for 30 min with 1% gluataraldehyde in 0.1 M cacodylate buffer pH 7.4. To visualize invadosomes, samples were stained for 2 h with phalloidin-TRITC (2.5 µg/ml). After several washes in cacodylate buffer the autofluorescence was quenched using 100 mM NH_4_Cl in 0.1 M cacodylate buffer for 10 min. Coverslips were then observed on an inverted microscope (Zeiss Axio Observer.Z1) using an Plan-NEOFLUAR 40× (NA 1.3) Oil objective. DAB precipitation was induced by illuminating the samples with a Xenon Lamp (TRITC filters) in the presence of an oxygenated solution containing 1 mg/ml of 3,3′-Diaminobenzidine tetrahydrochloride hydrate (D5637 SIGMA) in 0.1 M Cacodylate buffer until the fluorescent signal faded and a brown precipitate appeared (usually within 3–20 min). After the brown precipitate was fully developed the coverslips were removed from the microscope stage, washed with cacodylate buffer and directly postfixed with 1.5% of potassium ferrocyanide, 1% osmium tetroxide in 0.1 M cacodylate buffer, “*en bloc”* stained in 1% uranyl acetate in distilled water overnight at 4 °C and dehydrated in a graded series of ethanol solutions. Finally the samples were embedded in Epon812 resin and cured in an oven at 60 °C for 48 h. The cells of interest were localized in the resin block thanks to the brown DAB precipitate, a fragment of the resin block containing them was glued with cyanoacrylate glue on top of an empty Epon block and mounted on ultramicrotome (Leica FC7, Leica microsystem, Vienna, Austria). Ultrathin section (70–90 nm) were collected on formvar carbon–coated grids, stained with uranyl acetate and Sato’s lead solutions and observed in a Leo 912AB Zeiss Transmission Electron Microscope (Zeiss, Oberkochen, Germany). Digital micrographs were taken with a 2Kx2K bottom mounted slow-scan Proscan camera (*ProScan*, Lagerlechfeld, Germany) controlled by the EsivisionPro 3.2 software (Soft Imaging System, Münster, Germany).

For CLEM cells grown on gelatin-coated gridded MatTek dishes (MatTek Corporation, Ashland, MA) were fixed for 10 min with 4% paraformaldehyde, 0.05% glutaraldehyde in PBS. Cells were washed in PBS and treated with 50 mM NH_4_Cl for 10 min. Subsequently, cells were permeabilized with 0.1% saponin, and blocked in blocking buffer (0.2% gelatine in PBS) for 30 min. Cells were stained with TRITC-phalloidin and with anti-liprinα1 Abs and visualized with goat anti-rabbit Alexa 647 and goat anti-rabbit nanogold–conjugated secondary Abs (Nanoprobes, NY, USA) diluted in blocking buffer. After washes in PBS, cells were fixed in 1% glutaraldehyde and observed with an inverted microscope (Perkin Elmer UltraVIEW spinning disk confocal microscope with Plan-Apochromat 40× lens); the position coordinates of the cells of interest was annotated. Then nanogold was enlarged with gold enhancement solution (Nanoprobes, NY) according to manufacturer instructions, and cells were processed as for conventional EM. Ultrathin section (70–90 nm) were collected on formvar carbon coated slot grids, stained with uranyl acetate and Sato’s lead solutions and observed in a Leo 912AB Zeiss Transmission Electron Microscope (Zeiss). Digital micrographs were taken with a 2Kx2K bottom mounted slow-scan Proscan camera (ProScan, Lagerlechfeld, Germany) controlled by the EsivisionPro 3.2 software (Soft Imaging System, Münster, Germany). For relocating invadosomes and correlating liprin-α1 staining, EM images were aligned to fluorescent images using ec-CLEM Icy plugin and overlaid by means of Photoshop software. For quantification of the density of liprin-α1 in invadosome or perinuclear areas, the position of gold particles present in five align serial sections were recorded with ImageJ and visualized in pseudocolor (kernel density estimation done in Past statistic software ver. 3.14). For quantification of liprin-α1 distribution within each invadosomes 3 concentric areas (core, peripheral, and external) were defined, based on the actin core structure and the normalized density of gold particles (number of gold particles per µm^2^) was calculated using ImageJ (n = 22 invadosome profiles analyzed).

### Gelatin zymography

Gelatin zymography was performed as described^43^. NIH-Src cells were transfected with the indicated siRNAs for 24 h, washed with PBS, supplied with culture medium without serum, and processed after 44 h. Conditioned media were concentrated with centrifugal filters (Amicon Ultra, Milliopre, Billerica, MA) by spinning at 4550 g for 30 min at 4 °C. Cells were lysed in absence of protease inhibitors. Concentrated conditioned media and cell lysates were analysed for gelatin degrading activity by electrophoresis under non-reducing conditions on SDS-polyacrylamide (10%) gels containing 1 mg/ml gelatin type A (Sigma #1890). Gels were incubated for 30 min in renaturing solution (2.5% TritonX-100 in water), washed twice with water, incubated for 30 min at room T and subsequently for 18 h at 37 °C in developing buffer (50 mM Tris-HCl, pH 7.8, 200 mM NaCl, 5 mM CaCl_2_ and 0.02% Brij 35). White zones of lysis indicating gelatin degrading activity were revealed by staining with Coomassie brilliant blue.

### Statistical analysis

Significant differences were evaluated by χ^2^ test, Student t-test, or one-way ANOVA and post hoc Dunnet test. *p < 0.05; **p < 0.01; ***p < 0.001). All experiments were repeated at least two times and the data expressed as means ± s.e.m. (standard error of the mean).

## Electronic supplementary material

Supplementary video 1

Supplementary video 2

Supplementary video 3

Supplementary information
