# Supplementary material for: Identification of a membrane-less compartment regulating invadosome function and motility
Source: Sci Rep. 2018 Jan 18;8:1164. doi: 10.1038/s41598-018-19447-2 (PMC5773524; doi:10.1038/s41598-018-19447-2)
Supplement: Supplementary file 4 — Supplementary information [file 41598_2018_19447_MOESM4_ESM.pdf]

Supplementary Information

Identification of a membrane-less compartment regulating invadosome function and motility

Kristyna Sala, Andrea Raimondi, Diletta Tonoli, Carlo Tacchetti, Ivan de Curtis

Supplementary Figure Legends

Supplementary Figure 1. Quantification of the effects of siRNAs on ECM-degrading cells. (**A**) Immunoblot on lysates from MDA-MB-231 cells transfected with the indicated siRNAs. Percentage of ECM-degrading cells (**B**), and of cells with at least one invadosome (**C**); n=25-50 cells per experimental condition; *p<0.05, **p<0.01, ***p<0.001 vs siLuc; χ^2 test. (**D**) ECM degradation by cells cotransfected with control siRNA and FLAG-βGalactosidase (siLuc + βGal), with siRNA for liprin-α1 and FLAG-βGalactosidase (siLip + βGal), or with siRNA for liprin-α1 and FLAG-Liprin-α1 (human) that is siRNA–resistant (siLip + Lip-sr); n=69-71 cells from 2 experiments; ***p*<0.01 by the t-test.

Supplementary Figure 2. NIH-Src cells form ECM degrading invadosomes. (**A**) Immunoblotting on filters with lysates of NIH-3T3 and NIH-Src cells (18 μg of protein per lane). (**B**) Localization of Src and active phospho-Src at F-actin–positive invadosomes in NIH-Src cells. (**C**) In contrast to the fibroblast-like parental NIH-3T3 cells, NIH-Src cells (NIH-3T3 cells expressing the constitutively active kinase mutant c-Src-Y527F) form invadosomes that can degrade the ECM (6h on Oregon green-gelatin). Bars, 20 μm.

Supplementary Figure 3. Protein silencing by siRNAs and effects of siRNAs on invadosomes. (**A**) Immunoblot on lysates from NIH-Src cells transfected with the indicated siRNAs. (**B–C**) Quantification of protein levels in cells transfected with siRNAs for either one protein (**B**) or with multiple siRNAs for all proteins (**C**) were lysed and loaded on SDS-PAGE for immunoblotting (example shown in **A**). Bands of residual proteins were quantified and expressed as percentage of signal compared to control silencing (siLuc). Means ± s.e.m. from 5 (**B**) or 3 (**C**) experiments; t test, ***p<0.001, **p<0.01, *p<0.05. (**D–E**) Effect of protein silencing on the percentage of cells with invadosomes (**D**) or with actively degrading invadosomes (**E**). Bars are percentage of cells (n ≥100 cells for each condition, 3 experiments; *p<0.05, **p<0.01, ***p<0.001 vs control siLuc; χ^2 test.

Supplementary Figure 4. Endogenous protein levels in wild type and active Src–overexpressing NIH-3T3 cells. I mmunoblotting on lysates (18 μg protein/lane) from normal NIH-3T3 and transformed NIH-Src cells to compare the levels of proteins of the liprin complex in the two cell types. The levels of liprin-α1, ERC1 and LL5 proteins are similar in the two cell populations (graph on the right).

Supplementary Figure 5. Liprin-α1, ERC1, LL5 and paxillin do not accumulate near the invadopodia of invasive MDA-MB-231 cells. (**A**) Confocal imaging on MDA-MB-231 cells seeded for 18 h on 10ug/ml FN. (**B**) Cells were plated for 5 h on FN-coated Oregon–green gelatin before fixation and immunofluorescence for the endogenous liprin-α1, ERC1, LL5 proteins. The 4-fold enlargement shows a central area of cells with F-actin positive ECM degrading invadopodia. (**C**) Three-D reconstruction (XZ plane) from a stack of confocal images of a cell plated on Oregon–green gelatin. Arrowheads point to areas of ECM degradation corresponding to F-actin–positive invadopodia. Bars: 20 μm in **A**, **B**; 5 μm in **C**.

Supplementary Figure 6. Liprin-α1, ERC1 and LL5 are enriched near invadosomes of MDA-MB-231 expressing constitutively active Src-Y527F. (**A**) MDA-MB-231 cells transfected with Y527F Src for 24 h and plated on FN-coated Oregon–green gelatin for 5 h. Transfected cells form peripheral invadosomes that actively degrade ECM (arrows). The two images on the right are 3-fold enlargements of the area indicated by the arrows in the lower magnifications. (**B-D**) Src-Y527F transfected MDA-MB-231 cells plated for 18 h on 10 ug/ml FN were immunostained for endogenous liprin-α1 (**B**), ERC1 (**C**) or LL5 (**D**). The three proteins accumulate near invadosomes. Invadosomes are surrounded by paxillin–positive adhesion regions. The 4-fold enlargements on the right of each panel show the merges and single stainings of invadosomal areas indicated by the arrows in the lower magnifications. Bars, 20 μm.

Supplementary Figure 7.

Confocal images of A431 (**A-C**) and HT1080 cells (**D-G**), fixed and immunostained for the indicated endogenous proteins. Arrows indicate the same areas in the low magnification and in the corresponding enlargements. Bar: 20 μm.

Supplementary Figure 8. Overexpressed liprin-α1, ERC1 and LL5 accumulate near invadosomes. NIH-Src cells transfected with the indicated GFP-tagged constructs were fixed after replating for 18 h, and stained for F-actin (red) and the overexpressed protein (green = anti-GFP) for confocal microscopy.

Supplementary Figure 9. Effect of silencing on protein accumulation. Confocal images of NIH-Src cells cotransfected with GFP and the indicated siRNAs, and stained for F-actin (red), GFP (green) and either ERC1 (**A**), LL5 (**B**), or liprin-α1 (blue) (**C**).

Supplementary Figure 10. Original scans of filters for blots shown in Figure 2G and L. Blots from gels loaded with lysates of cells treated with the indicated siRNAs were blotted with antibodies for the indicated proteins.

Supplementary Figure 11. Depletion of liprin-α1, ERC1a or LL5 proteins affects the formation of invadosomes. (**A**) Quantification of the accumulation of liprin-α1, ERC1 and LL5 proteins after depletion of either protein. Bars are mean values ± s.e.m. of the accumulation of the endogenous proteins near invadosomes following silencing of the other proteins of the liprin complex; n=69-154 invadosomes from 16-28 cells from 2-3 experiments; ANOVA, Dunnet post hoc, ***$p<0.001$ vs siLuc. (**B-D**) Quantification of the effects of protein silencing on invadosomes. NIH-Src cells silenced for 48 h with the indicated siRNAs and GFP were replated for 18 h on coverslips. (**B**) representative cells transfected with the indicated siRNAs; only the staining for F-actin is shown. (**C**) The percentage of cells with invadosomes was not affected by silencing of either protein. (**D**) Quantification of the cell area, number of invadosomes per cell, percentage of cell area occupied by invadosomes, and mean invadosome area (n = 76-113 cells from two experiments). Graph bars on the right (Mean invadosome area) were obtained by averaging the mean invadosome areas of each cell. ANOVA, Dunnet post hoc, *p<0.05, **p<0,01, ***p<0,001 vs siLuc.

Supplementary Figure 12. Liprin-α1, ERC1 and LL5 proteins are not required for the localization of MT1-MMP at invadosomes. (**A**) Distribution of endogenous MT1-MMP in NIH-Src and control NIH-3T3 cells. Colocalization of MT1-MMP with F-actin and cortactin at invadosomes in NIH-Src cells. (**B**) Colocalization of endogenous MT1-MMP with F-actin at ECM degrading invadosomes. NIH-Src cells plated for 5 h on Oregon–green gelatin. (**C-E**) NIH-Src cells transfected with siRNAs for luciferase, liprin-α1, or cotransfected with siRNAs for liprin-α1, ERC1 and LL5 proteins (siAll) were replated for 1 h on gelatin. (**C**) Confocal imaging on transfected cells (red) showing the distribution of invadosomes (F-actin, green) and MT1-MMP (blue) in cells transfected with indicated siRNAs. (**D**) Immunoblotting on cell lysates from the cell preparation used in (**C**). (**E**) Quantification of the accumulation of MT1-MMP at invadosomes. Bars are mean ratios ± s.e.m. of MT1-MMP integrated density at invadosomes divided by the area of phalloidin-positive invadosomes (23-25 cells/condition). ANOVA, Dunnet post hoc.

Supplementary Figure 13. Gelatin zymography was performed on non-reducing SDS-PAGE with gelatin, loaded with 40 μg/lane of protein from cell lysates (bottom) or from concentrated media (top).

Cleared bands visible after coomassie staining indicate the position of gelatine degradation by metalloproteases.

Supplementary Figure 14. (**A**) Nocodazole–induced depolymerization of microtubules does not affect the localization of endogenous LL5, ERC1 and liprin-α1 near invadosomes. Treated cells were immunostained with phalloidin and Abs for LL5 and ERC1 (***a***), or with Abs for tubulin and liprin-α1 (***b***). (***c, d***) The distribution of microtubules is not evidently affected by silencing of liprin-α1. (**B**) Immunoblotting with anti-Src, anti-pSrc and anti-calnexin Abs on lysates from NIH-Src cells lysed before treatment (T_0), after 60 min incubation with 10 μM PP2/DMSO or with DMSO alone (T_{60}), or after treatment followed by 10 min rescue in absence of inhibitor (T_{60}+R_{10}). The same filter was probed first for pSrc and then reprobed after stripping for Src. For blotting, 20 μg of protein lysate were loaded on each lane of a 7.5% acrylamide SDS-PAGE; t test: p=0.012 (PP2 T_{60} vs DMSO T_{60}), p=0.0022 (PP2 T_{60} vs T_0).

Supplementary Figure 15. Graph with the percentage of cells with high or low Src levels, as detected by immunofluorescence with anti–Src Abs. The ratio between the percentage of cells with high or low Src levels was not affected by inhibitors and by silencing of liprin-α1 protein (n=186-309 cells, 3 experiments).

Supplementary Figure 16. NIH-Src cells co-transfected with siRNAs and GFP-LifeAct (invadosomes), fixed before (**A**, T_0) or after incubation with nocodazole and PP2 (**B**, T_{60}), and after washout and incubation for 5 min to rescue invadosomes (**C**, R_5). Immunostaining to reveal GFP-LifeAct (green), Src (red), and phosphoSrc (blue). Bars, 20 μm.

Supplementary Figure 17. Panels **a–c**: confocal images of cells stained for F-actin and endogenous liprin-α1. Clustering of liprin-α1 at F-actin–positive invadosomes is evident. Panels **d** to **e**: TEM micrograph of the invadosome shown by an arrow in **a-c**. In **d** is shown the elctron micrograph only; in **e** is shown the overlay of the fluorescent signal (in color) on the TEM micrograph. Scale bar, 1.5 μm. Immunofluorescence (**f**) and low magnification electron microscopy on two thin sections (**g, h**) of the same cell shown in (**f**). Dotted rectangles show the same invadosomal area in the three images.

Supplementary Movie Legends

Supplementary Movie 1. NIH-Src cells co-transfected with GFP–Liprin-α1 and mCherry–ERC1. Time-lapse with a Leica TCS SP8 laser scanning confocal microscope equipped with 63x. Frames every 10 sec, for 15 min.

Supplementary Movie 2. NIH-Src cells co-transfected with GFP–LifeAct and mCherry–ERC1. Time-lapse with a Leica TCS SP8 laser scanning confocal microscope equipped with 63x. Frames every min, for 60 min.

Supplementary Movie 3. NIH-Src cells co-transfected with GFP–LifeAct and siLuc. Time-lapse with a Leica TCS SP8 laser scanning confocal microscope equipped with 63x. Frames every 3 min, for 180 min.

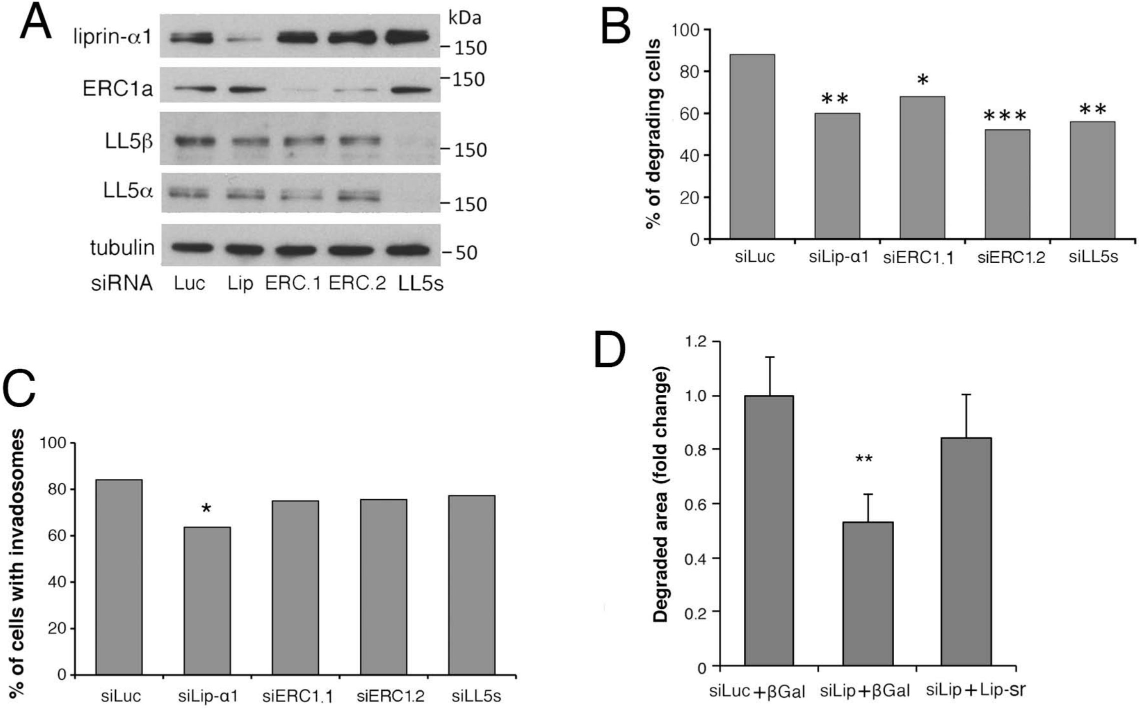

Supplementary Figure 1

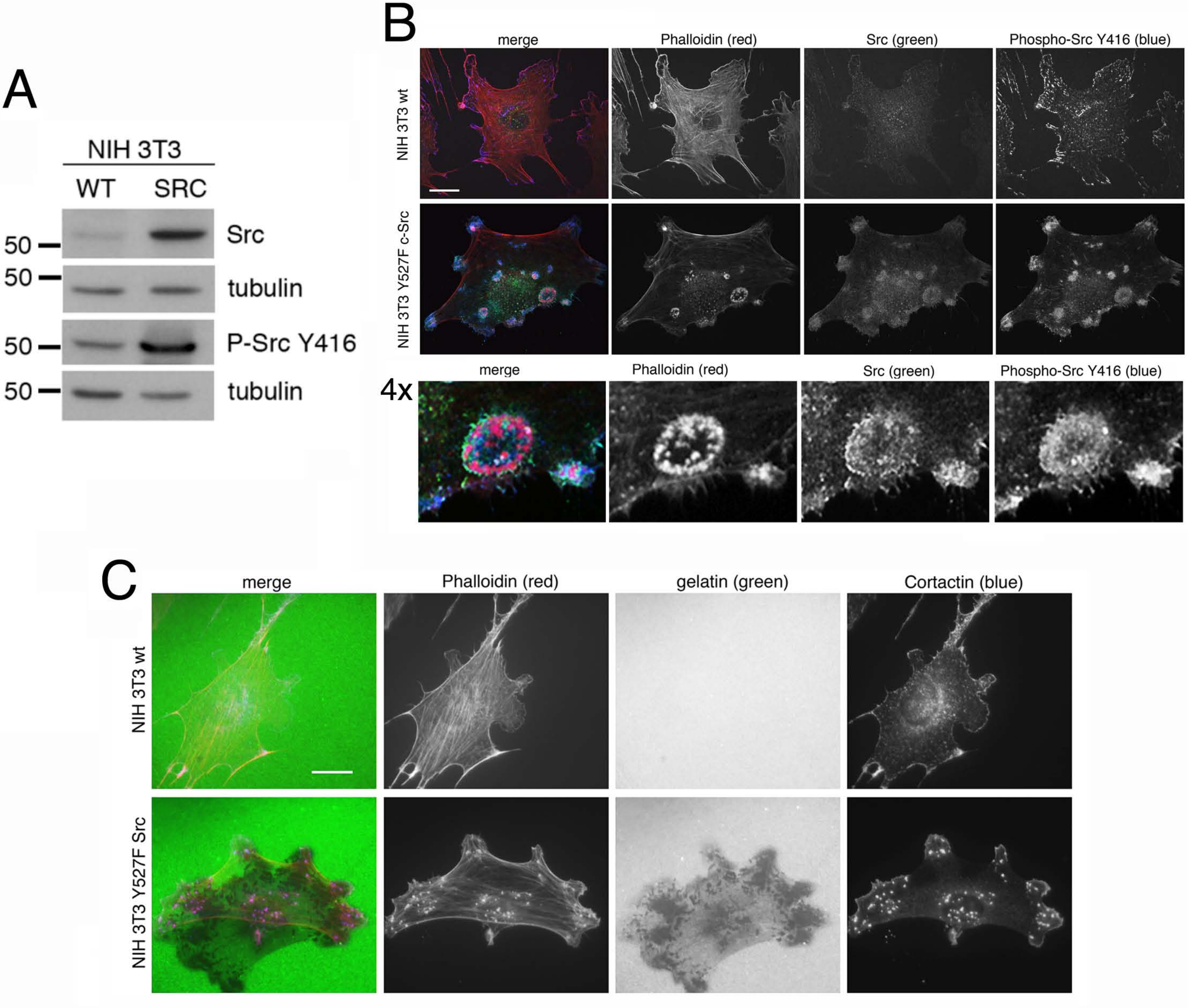

Supplementary Figure 2

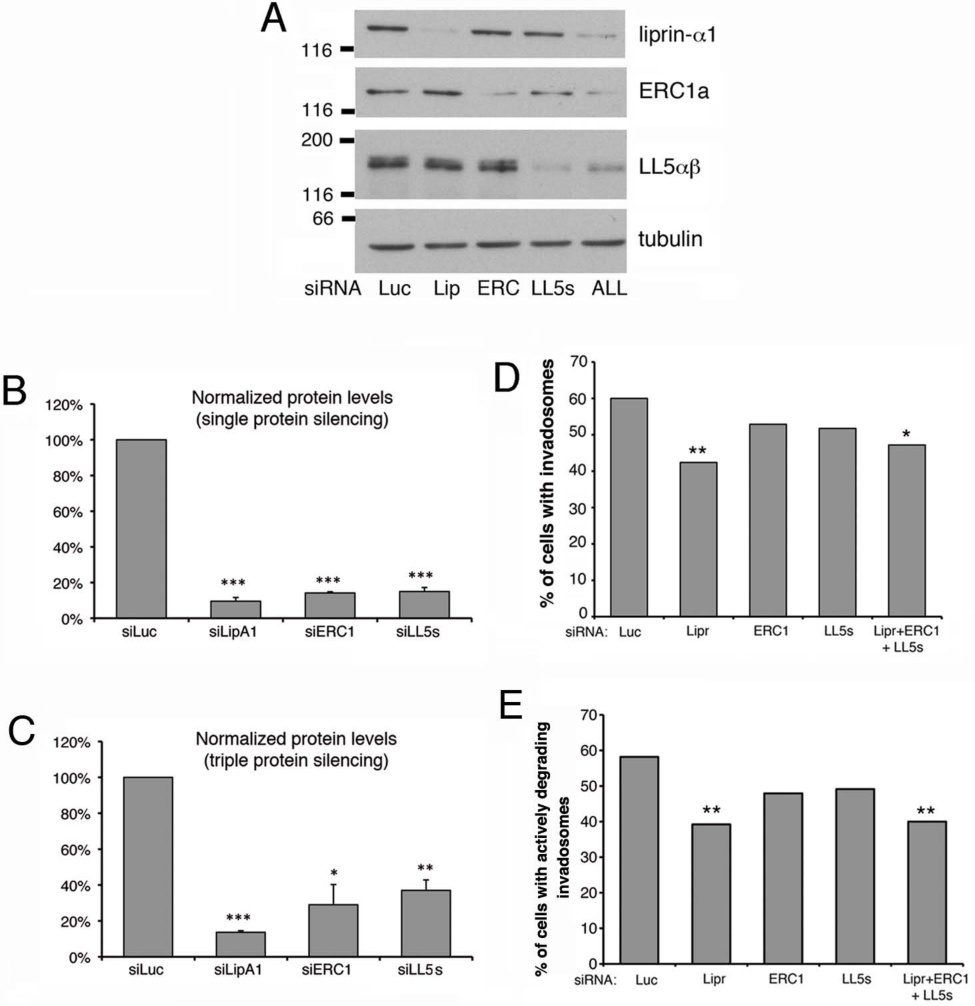

Supplementary Figure 3

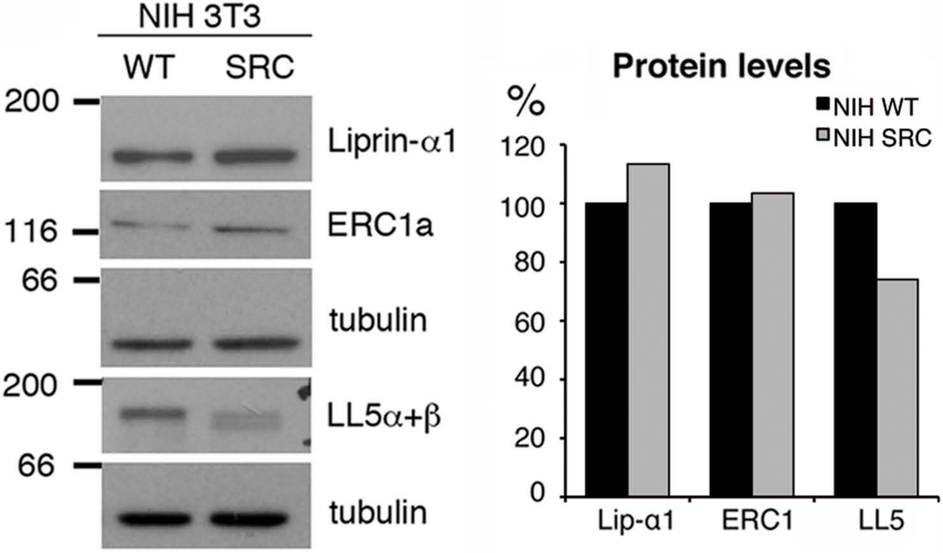

Supplementary Figure 4

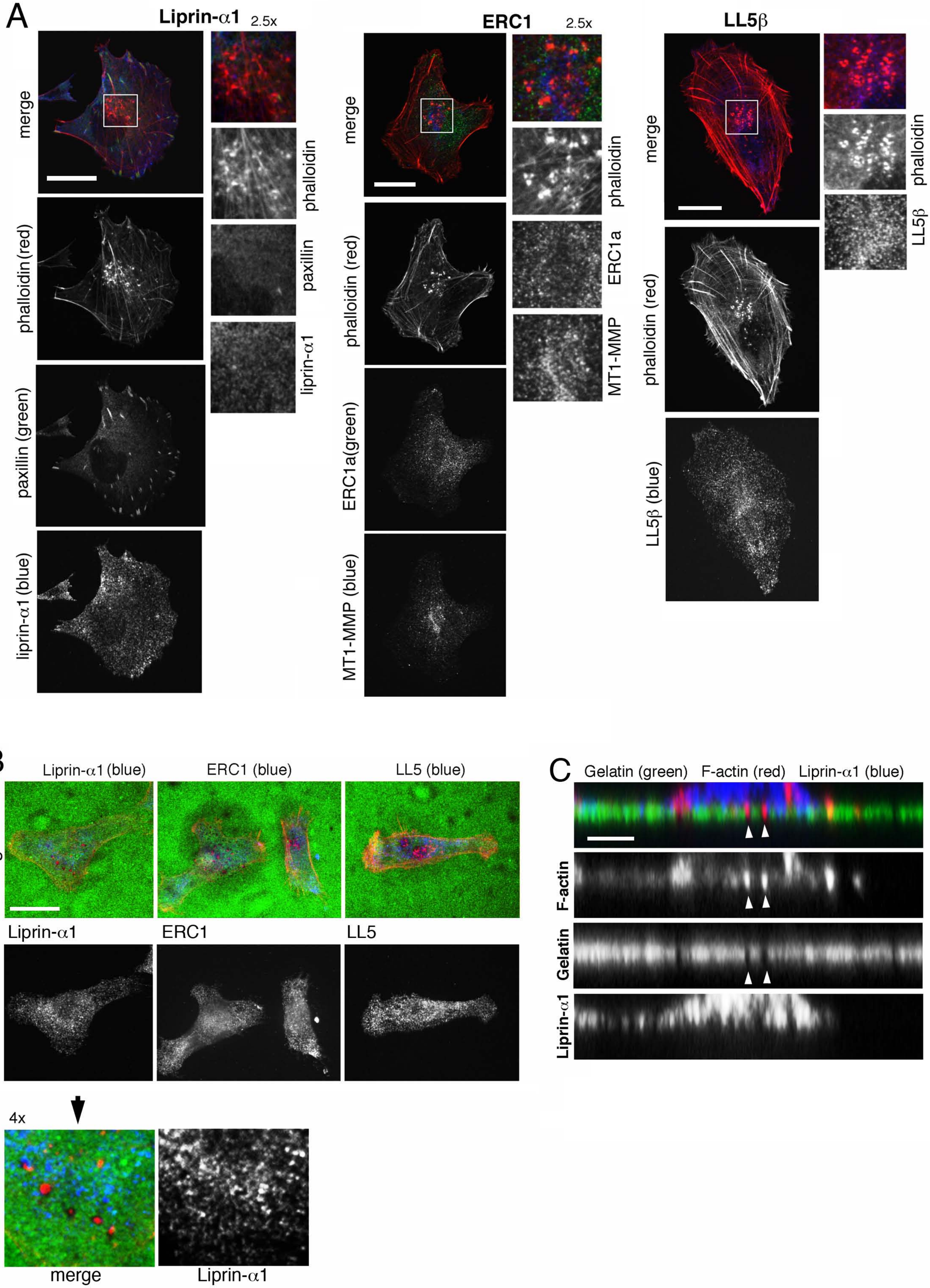

Supplementary Figure 5

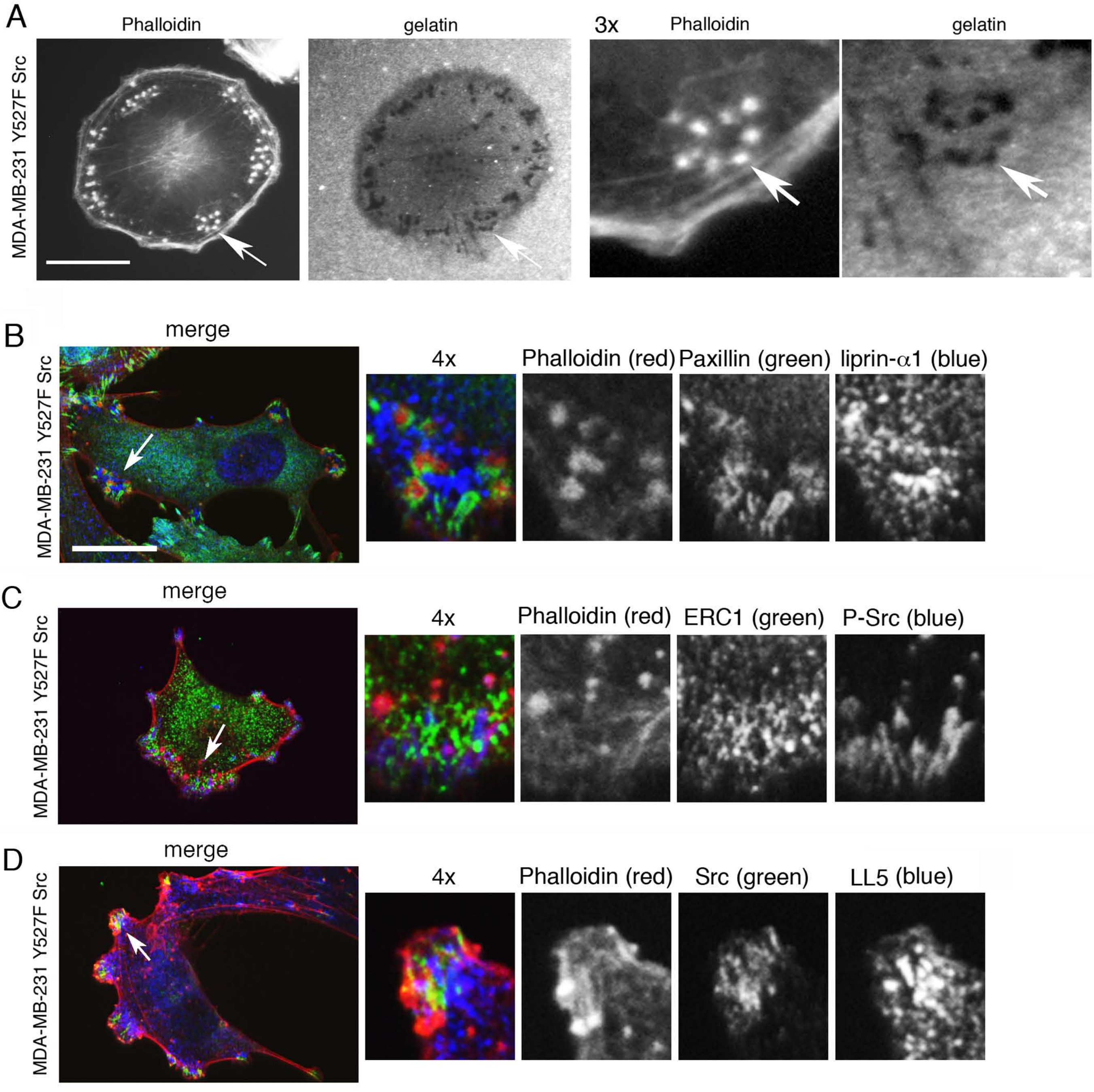

Supplementary Figure 6

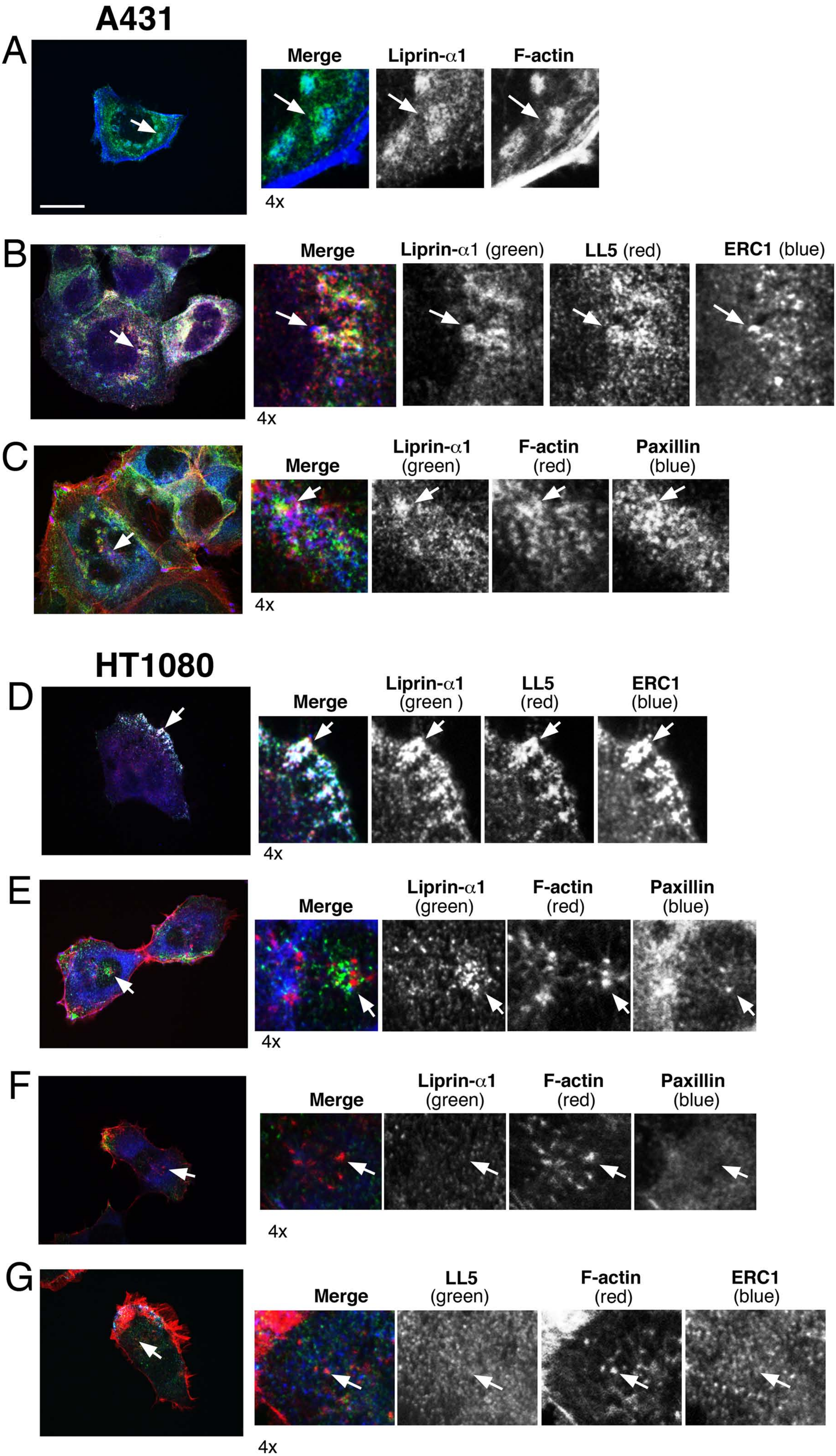

Supplementary Figure 7

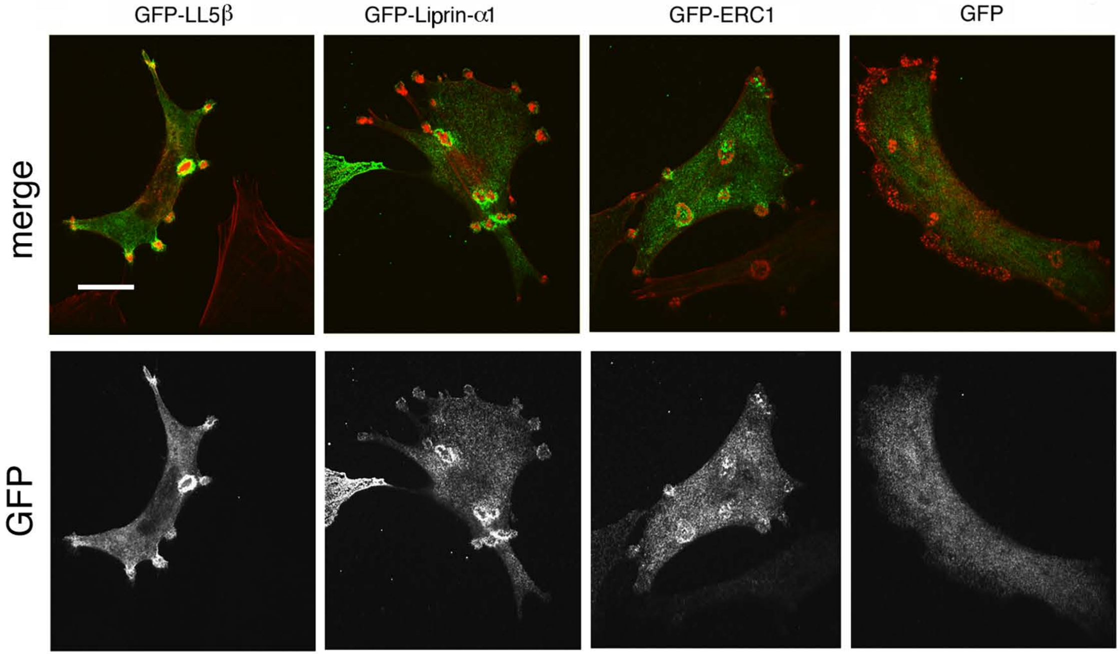

Supplementary Figure 8

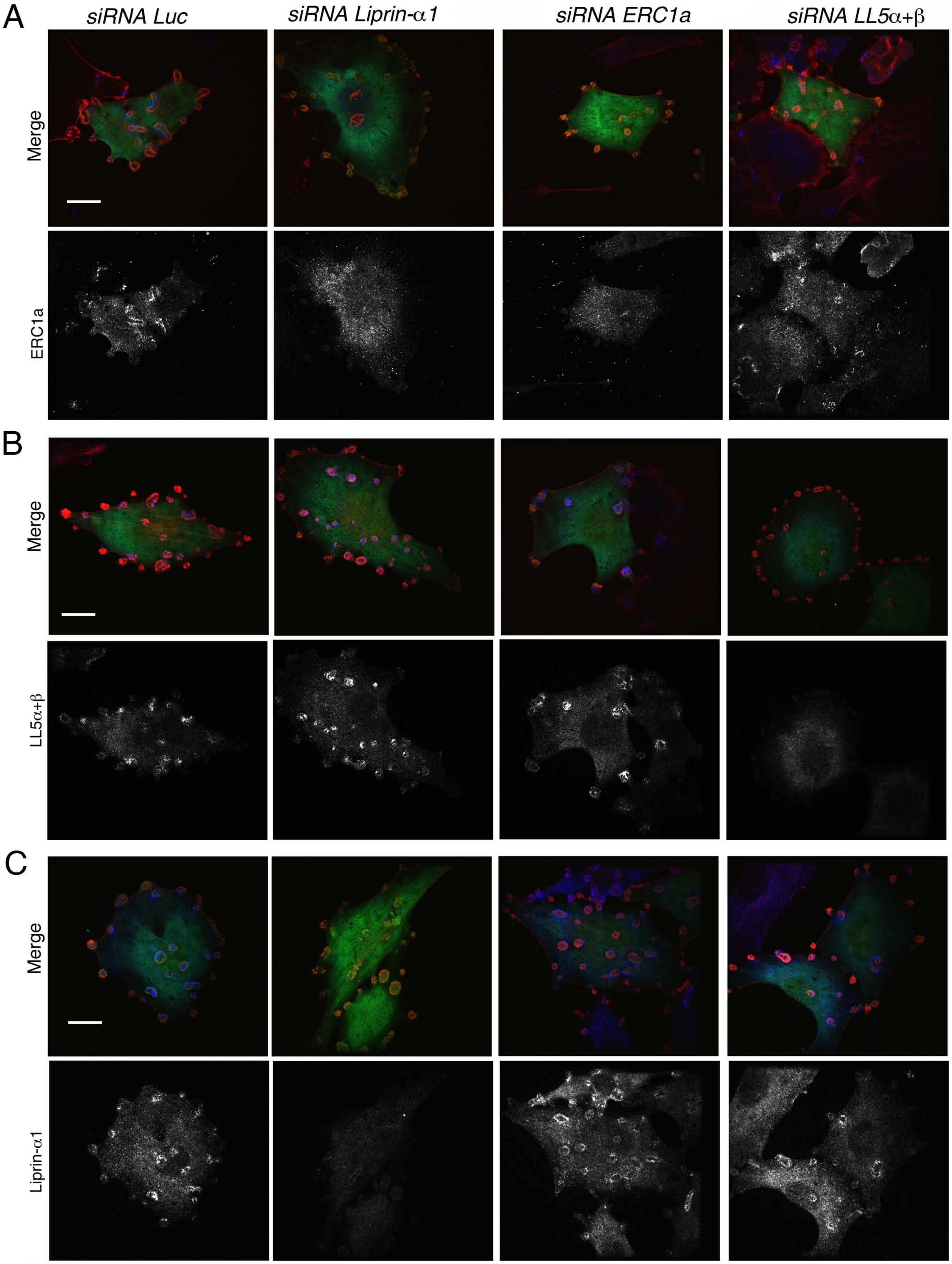

Supplementary Figure 9

Filters for blots shown in Figure 2G

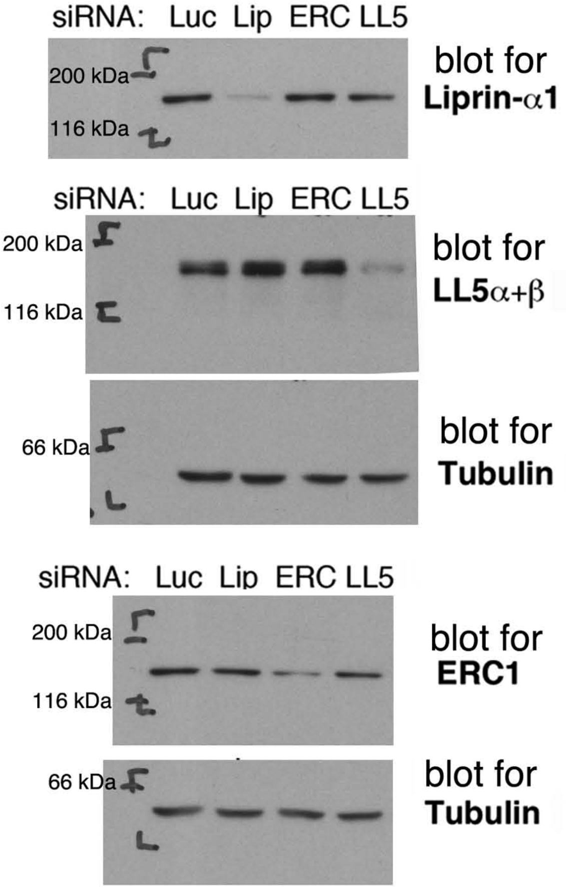

Filters for blots shown in Figure 2L

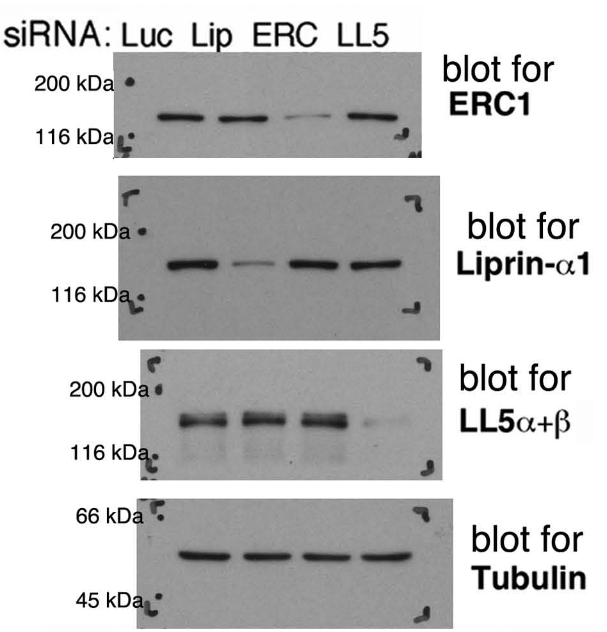

Supplementary Figure 10

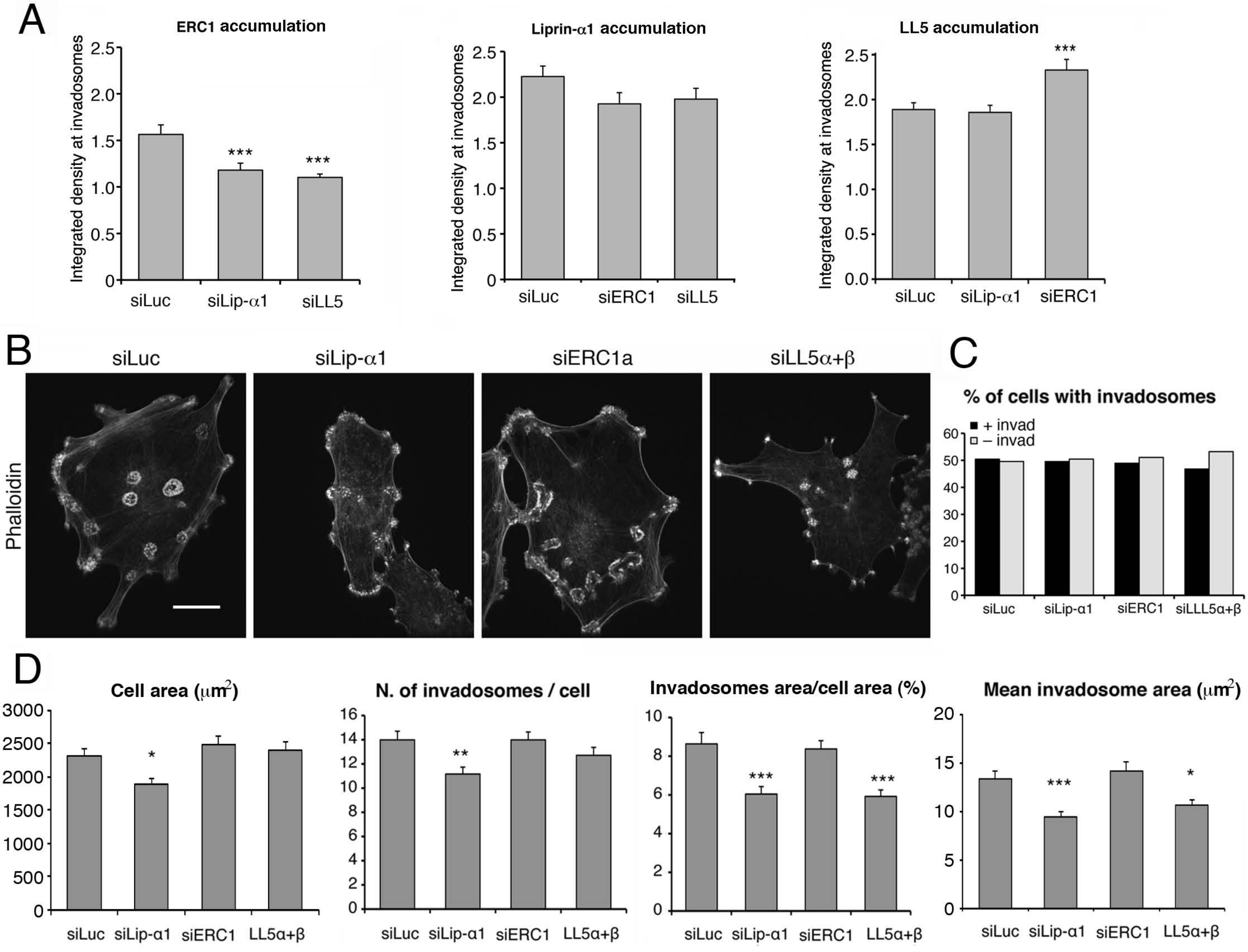

Supplementary Figure 11

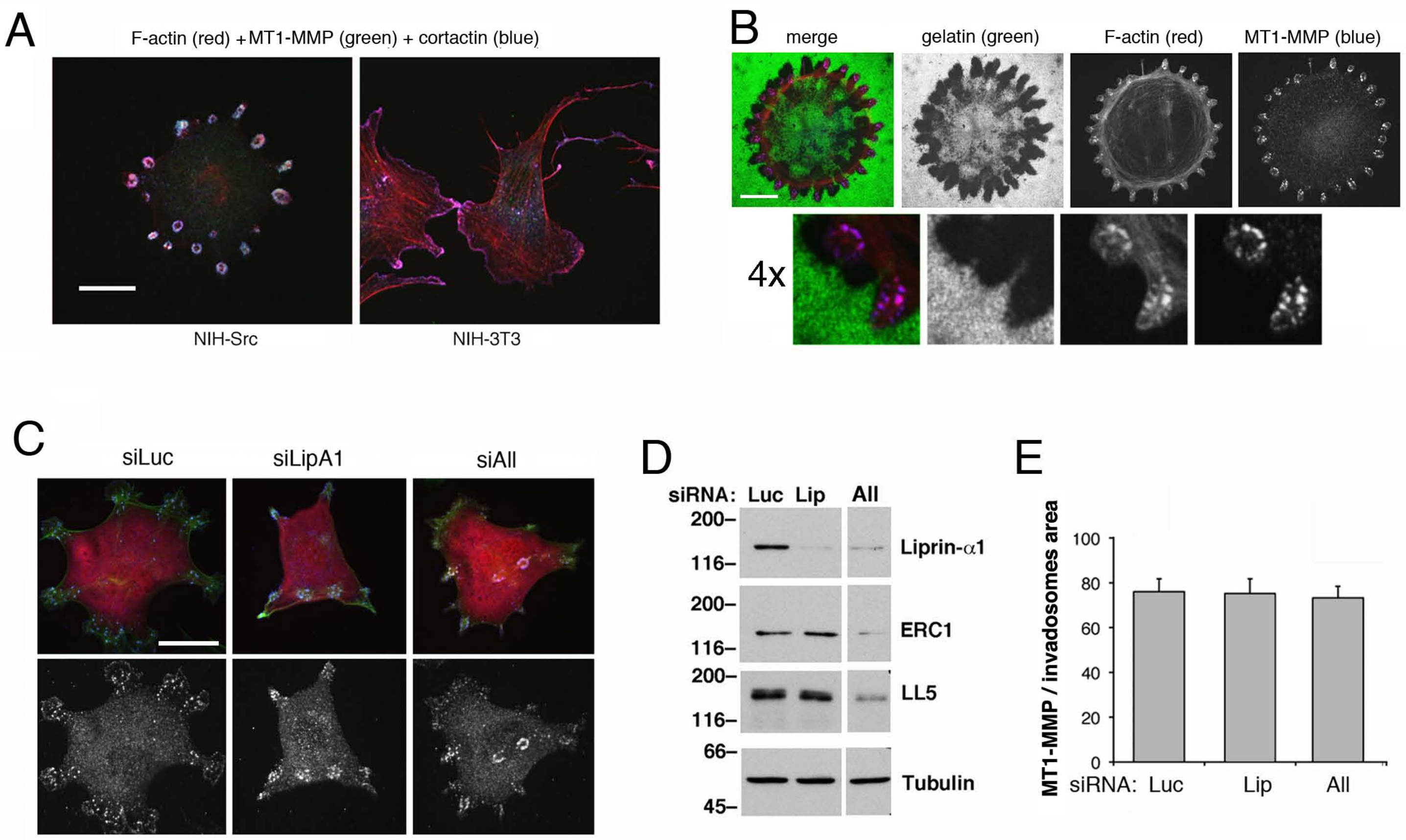

Supplementary Figure 12

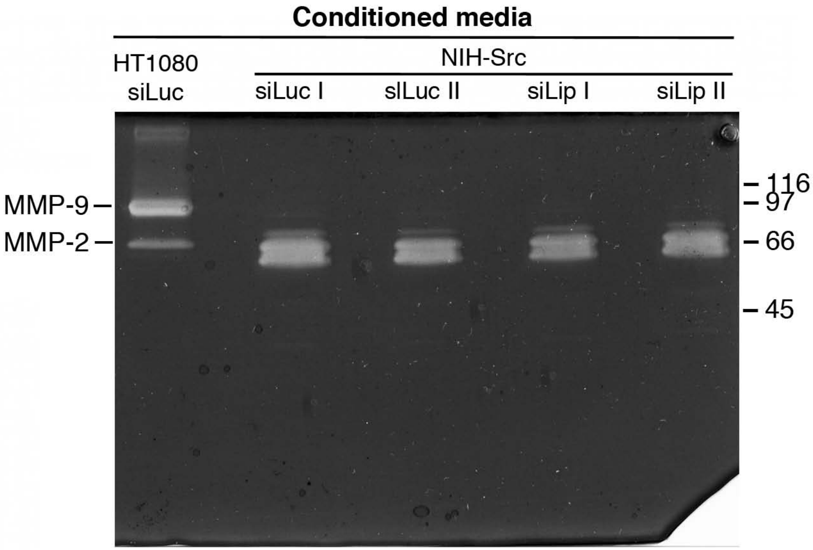

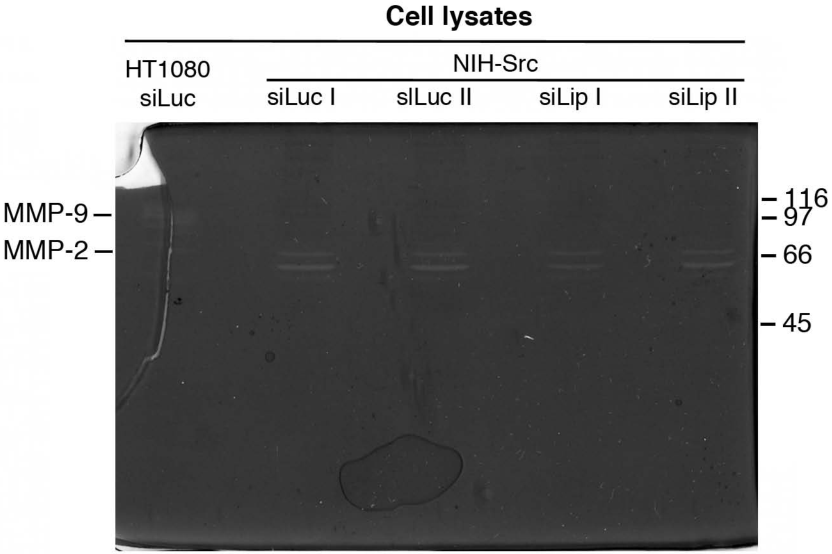

Supplementary Figure 13

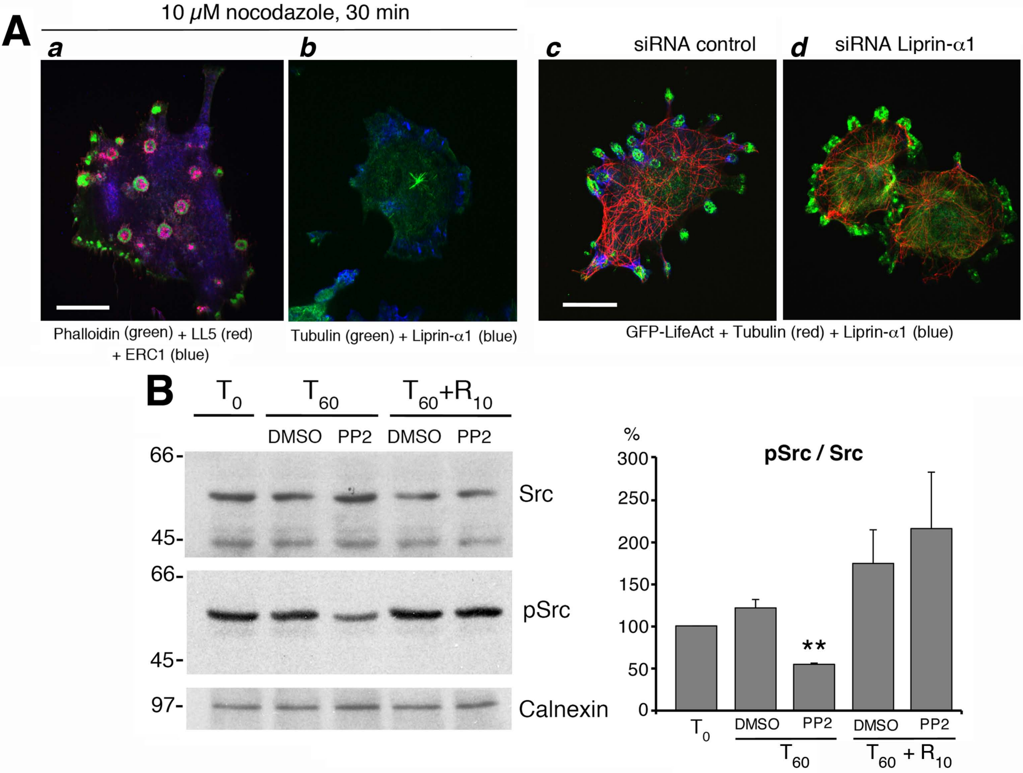

Supplementary Figure 14

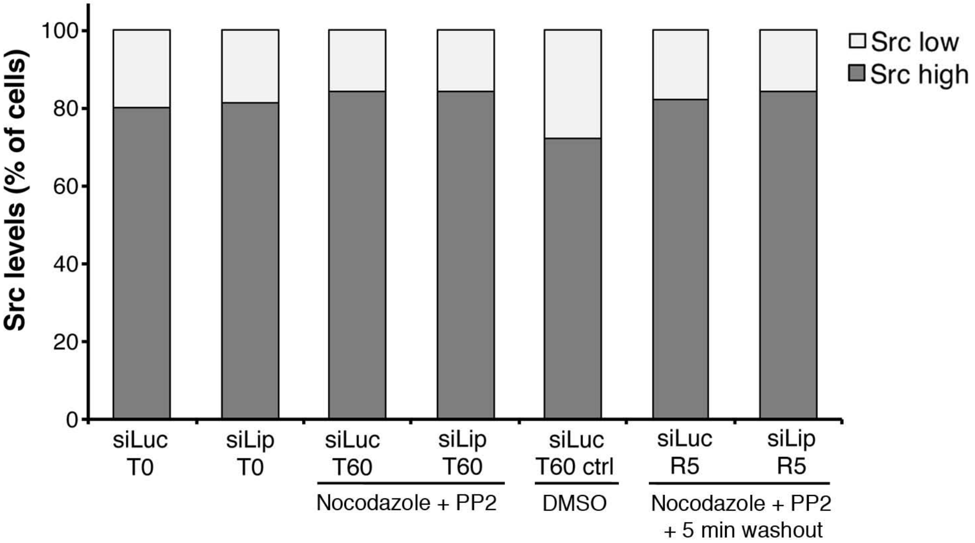

Supplementary Figure 15

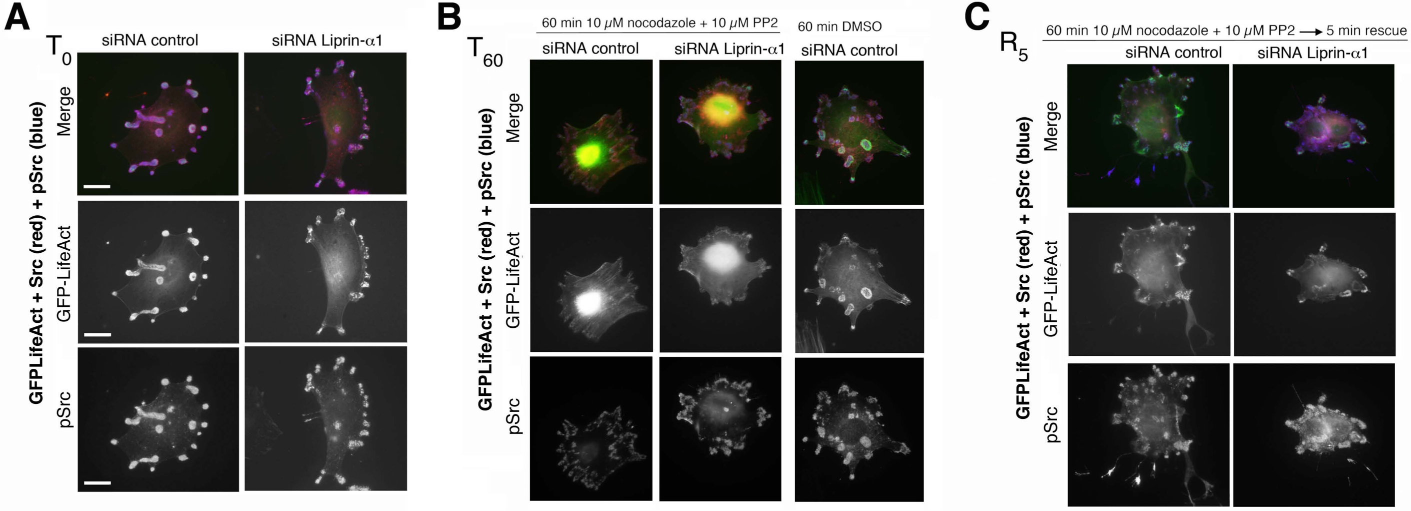

Supplementary Figure 16

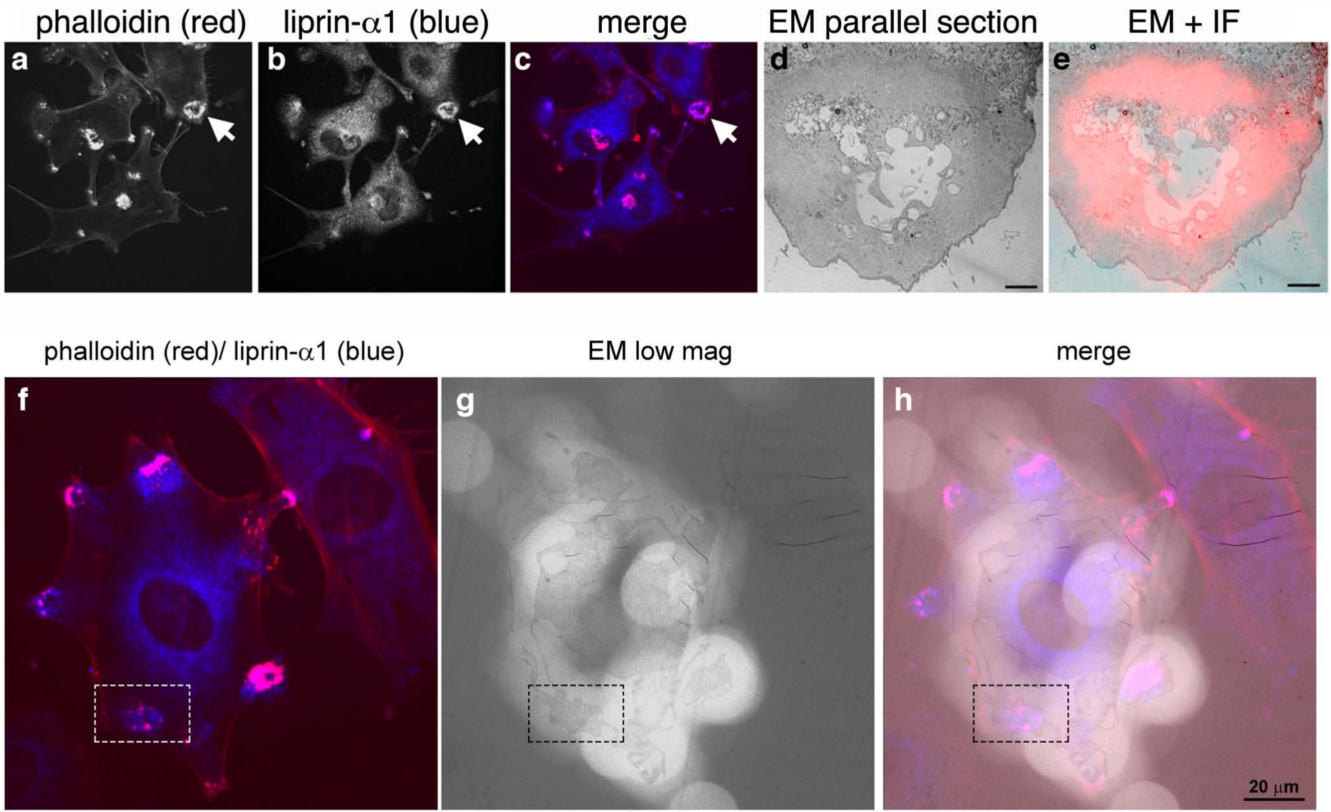

Supplementary Figure 17
